# Study and exploration of the pharmacokinetics of traditional Tibetan medicine Ruyi Zhenbao tablets after single and long-term administration

**DOI:** 10.3389/fphar.2022.948693

**Published:** 2022-09-29

**Authors:** Hongping Hou, Tengfei Chen, Ziying Xu, Zihui Yu, Caixia Wang, Rongxia Liu, Bo Peng, Wei Yang, Feng Li, Xiangyi Che, Bing Li, Yu Wang, Ling Song, Yunhang Gao, Zuguang Ye, Guangping Zhang

**Affiliations:** ^1^ Institute of Chinese Materia Medica, China Academy of Chinese Medical Sciences, Beijing, China; ^2^ Capital Institute of Pediatrics, Beijing, China; ^3^ School of Pharmacy, Yantai University, Yantai, China; ^4^ Gansu Cheezheng Tibetan Medicine Co., Ltd., Beijing, China; ^5^ Yantai Saipute Analyzing Service Co., Ltd., Yantai, China

**Keywords:** Tibetan medicine, Ruyi Zhenbao tablet, single administration, long-term administration, pharmacokinetics

## Abstract

Tibetan medicine is one of the oldest traditional medicine systems in the world. Taking the Ruyi Zhenbao tablet (RYZB) as an example, which is a widely used classic oral Tibetan medicine, this article discusses the pharmacokinetics of single administration and long-term treatment and analyzed its metabolic properties and tissue distribution *in vivo*. After single administration, blood samples were collected before administration and at different time points after administration in different groups of rats. In the study of long-term treatment effects, blood samples were collected from the animals in each group on days 1, 15, and 30 and on day 15 after withdrawal. The results showed that after a single administration, the dose change had no significant effect on the T_1/2_ and T_max_ of agarotetrol, isoliquiritigenin, and piperine (*p* > 0.05). There was a certain correlation between the increase in AUC_0-t_ and the C_max_ of agarotetrol, isoliquiritigenin, piperine, and the increase in dosage, with a dose range of 0.225–0.900 g/kg. There were no significant differences in C_max_ and AUC_0-t_ of ferulic acid at different doses (*p* > 0.05). Meanwhile, there was no significant sex-based difference in the pharmacokinetic parameters of these four components in rats. After long-term administration, the distribution agarotetrol in various tissues of rats was kidney > liver > heart > brain; the tissue distribution in low- and medium-dose groups of isoliquiritigenin was liver > kidney > heart > brain, and in the high-dose group, kidney > liver > heart > brain. The tissue distribution of piperine in each dose group was liver > kidney > heart > brain, and that of ferulic acid in each dose group was kidney > liver > heart > brain. Through the establishment of the previously developed methodology, the pharmacokinetic properties of RYZB were analyzed after a single administration and long-term administration. Our findings confirmed this approach for the exploration and establishment of a pharmacokinetic evaluation of Tibetan medicine, to support its guiding role in clinical application, but also to accelerate research into Tibetan medicine theory and medicine and to provide a solid foundation for the translation of Tibetan medicine throughout the world.

## Background

Tibetan medicine, originating from Tibet, China, is one of the oldest traditional medicine systems in the world. In its natural environment with high altitude, thin air, and lack of oxygen, through long-term rich production and living practice, Tibetan medicine has unique properties and advantages over other traditional medicines, generating a complete medical theoretical system with good clinical efficacy (Dakpa, 2014; Sun et al., 2020), and thus, has gradually been accepted throughout China and across the world. However, basic research on Tibetan medicine has been relatively limited and no accurate clinical guidelines or rationale drug usage standards have been established (Liang et al., 2021). Therefore, analyzing the characteristics and advantages of classical Tibetan medicine using modern medical techniques and means can not only support its clinical value in modern medicine but may also complement the shortcomings of modern medicine (Guo et al., 2012).

Ruyi Zhenbao tablet (RYZB) is a widely used classic oral Tibetan medicine, which was recorded in the book Tibetan Medicine Notes written by the famous doctor Gong Zhi Yuan Dan Jia Cuo. RYZB consists of 30 types of medicinal constituents, such as *Mother-of-Pearl*, *Aquilariae Lignum Resinatum*, *Travertine*, *Micae Aureus Lapis*, *Carthami Flos*, *Brachyura*, *Caryophylli Flos*, and *Fructus Terminaliae Billericae*. It has effects of clearing away heat, resuscitating, relaxing muscles and tendons, activating collaterals, and drying yellow water, and has mainly been used to treat diseases such as white chanel disorder (a term of Tibetan medicine, which means diseases of the nervous system in modern medical science), numbness of the limbs, paralysis, facial distortion, unconsciousness, arthralgia, gout, limb rigidity, and joint disadvantage (Liu et al., 2015; Wang et al., 2016).

In the previous pre-experiment, we did a lot of work to explore the pharmaceutical ingredients in RYZB that need to be detected. Different from chemical medicine, RYZB contains a lot of medicinal materials and there are many components in different medicinal materials. At present, pharmacokinetic research on TCM was very little, especially in Tibetan medicine. The current research studies on RYZB mainly focused on pharmacology and quality standards (Liu et al., 2015; Gongbao et al., 2019; Ling et al., 2022), but there were no reports on related pharmacokinetics.

Thus, in this study, based on the composition of medicinal materials in RYZB and the prior studies on blood components, the main active components in the medicinal materials from RYZB, which were successfully detected in plasma and were more suitable for the construction of analytical methods; meanwhile, whose standard products could be purchased, were used for the pharmacokinetic study (Wang et al., 2014; Xu et al., 2018; Guo et al., 2020). A total of 10 standard materials (4,4′-dihydroxy-2-methoxychalcone, thymoquinone, daidzein, quercetin-3-rhamnoside, chlorogenic acid, agarotetrol, piperine, ferulic acid, cholan-24-oic acid, isoliquiritigenin, vestitone, and gallic acid) were purchased and used to screen for the target compounds. Finally, agarotetrol (C_17_H_18_O_6_) present in *Aquilariae Lignum Resinatum*, piperine (C_17_H_19_NO_3_) in *Piper longum* L., isoliquiritigenin (C_15_H_12_O_4_) in *Dalbergia odorifera* T. Chen, and ferulic acid in licorice cream were selected.

Now we used RYZB as a typical example, to evaluate the pharmacokinetic characteristics of single administration and long-term administration of a traditional Tibetan medicine in detail. The metabolic characteristics, tissue distribution, and deposition *in vivo* were analyzed (EMEA, 1994; FDA, 2001) to determine the characteristics of the medication rules of Tibetan medicine and to demonstrate the accurate clinical role and rationale for the use of RYZB within the practice of Tibetan medicine.

## Materials and methods

### Reagents and drug information

RYZB was purchased from Gansu Cheezheng Tibetan Medicine Co., Ltd., China, batch no. 2004007 (date of manufacture, April 2020; date of expiration, April 2023). Agarotetrol was obtained from Chengdu Push Biotechnology Co., Ltd., China, batch no. PU0295-0025, purity 99.7%. Piperine was obtained from Dalian meilunbio^®^ Co., Ltd., China, batch no. PU0295-0025, purity 99.0%. Isoliquiritigenin was purchased from Shanghai Macklin Biochemical Co., Ltd., China, batch no. C10987045, purity 99.2%. Ferulic acid was purchased from Shanghai Macklin Biochemical Co., Ltd., China, batch no. C11347527, purity 99.2%. Vorinostat was obtained from Shanghai Macklin Biochemical Co., Ltd., China, batch no. C11120592, purity 98.7%. Methanol (chromatographic purity) was purchased from Merck, United States, batch no. l1108107033 and formic acid amine was from Sinopharm Chemical Reagents Co., Ltd., China, batch no. 30011661.

### Equipment

The liquid chromatography system, Waters ACQUITY UPLC H-CLASS ultra-high performance liquid chromatography, was obtained from Waters Company, United States. High-resolution mass detection was performed on a Q-Exactive-Orbitrap mass spectrometer, Thermo Fischer Scientific, United States; mass spectrometry system, 6500+ triple quadrupole tandem mass spectrometer, SCIEX Company, United States. The analytical balance was obtained from the METTLER TOLEDO XPE205 millionth analytical balance, Mettler Toledo Instrument Co., Ltd., United States and the centrifuge, EPPENDORF 5427R small desktop freezing high-speed centrifuge was from EPPENDORF Company, Germany. The vortex mixer, IKA VORTEX2, was from IKA Company, Germany.

### Experimental methods

#### Single administration

Clinical dosage of RYZB: each tablet weighs 0.5 g, 4–5 tablets once, twice a day (Schultze-Mosgau et al., 2022). The clinical dosage for human is 4–5 g. According to the multiple body surface areas of humans and rats, the equivalent dose for rats is 0.450 g kg^−1^. A total of 24 SD rats (180–220 g, 3–4 weeks old) were randomly divided into four groups: control group, low-dose group (2× equivalent dose, 0.225 g kg^−1^), middle-dose group (equivalent dose, 0.450 g kg^−1^), and high-dose group (1/2 equivalent dose, 0.900 g kg^−1^), at a ratio of 1:1 males to females. Blood samples were collected from the eye socket before and at 0.25, 0.5, 0.75, 1, 2, 4, 8, 24, and 48 h after administration and heparin anticoagulation was used. Samples were centrifuged at 4°C and 10,000 rpm for 2 min, divided into aliquots, and frozen at −80°C.

#### Long-term administration

##### Plasma analysis

A total of 64 SD rats (180–220 g, 3–4 weeks old) were randomly divided into four groups: control group, high-dose group (maximum administration concentration, 6.0 g kg^−1^), middle-dose group (1/2 of high dose, 3.0 g kg^−1^), and low-dose group (1/2 of middle dose, 1.5 g kg^−1^), with 16 rats in each group, divided into eight males and eight female rats, of which eight rats were treated at 1 month after administration (Geisser and Banke-Bochita, 2010). The remaining eight animals were treated and collected half a month after withdrawal. Blood samples were collected from the eye socket of each group on the day of administration (D1), the middle period of administration (D15), and the last administration (D30) at 0.25, 0.5, 0.75, 1, 2, 4, 8, and 24 h after administration, and blood was collected 15 days after administration. Blood samples were processed as indicated previously.

##### Tissue measurement

One month after drug administration and 15 days after drug withdrawal (defined the recovery period), the animals were anesthetized and blood was drawn from the abdominal aorta (Bo and Wu, 2020). Organs such as the heart, liver, kidney, and brain were collected, washed quickly with saline at 4°C, weighed, and frozen at −80°C until use.

### Treatment methods and processing of plasma samples

#### Pre-treatment of samples

A 50.0 µl volume of rat plasma was placed in a 2.0 ml centrifuge tube and 150 µl of methanol solution (80.0 ng ml^−1^) containing internal standard was added (Polson et al., 2003; Pang et al., 2022). The solution was swirled for 1 min and then centrifuged for 15 min (12,700 rpm, 4°C). A 100 µl volume of supernatant was transferred to a 2.0 ml centrifuge tube and diluted with 50.0 µl of water. The sample was swirled for 1 min to mix and 100 µl of supernatant was transferred to a sample vial and then analyzed with LC-MS/MS.

#### Preparation of the standard curve and quality control of rat plasma

A 45.0 µl sample of blank rat plasma was transferred into a 2.0 ml centrifuge tube and 5.0 µl of standard series solution or of quality control solution was added (Janssen et al., 2022). After swirling for 10 s, plasma concentrations of piperine and isoliquiritigenin were equal to 0.100, 0.500, 1.000, 4.000, 10.000, 40.000, 100, and 200 ng ml^−1^, respectively. The concentrations of agarotetrol and ferulic acid were equivalent to 0.500, 2.500, 5.000, 20.000, 50.000, 200, 500, and 1,000 ng/ml or that of isoliquiritigenin and piperine was equivalent to 0.300, 1.500, 15.000, and 150 ng/ml. The concentrations of agarotetrol and ferulic acid were equivalent to 1.500, 7.500, 75.000, and 750 ng ml^−1^. LC-MS/MS analysis was carried out.

#### Analysis conditions

##### Chromatographic conditions

Chromatographic column: waters ACQUITY UPLC BEH C18, 2.1 mm × 100 mm I.D, 1.7 μm (Waters Corporation, United States); precolumn: ACQUITY UPLC BEH C18 VanGuard Pre-column, 130 Å, 1.7 µm, 2.1 mm × 5 mm; mobile phase A (MPA): 5 mmol L^−1^ ammonium formate aqueous solution (containing 0.1% formic acid); mobile phase B (MPB): methanol; column temperature: 4°C; flow rate: 0.300 ml min^−1^; temperature of the automatic sampler: 4°C; injection volume: 5.00 μl; the chromatographic gradients are described in Supplementary Table S1 (Jiang et al., 2010).

##### Mass spectrometry

The following mass spectrometry conditions were used: ion source: electrospray ionization (ESI); ionization mode (positive/negative); mode: multi-reaction monitoring (MRM); ion spray voltage: 5,500–4,500 V; turbo ion spray temperature: 450°C; curtain gas type: nitrogen setting, 35.0 psi; nebulizing gas; gas 1: nitrogen setting, 50.0 psi; auxiliary gas, gas 2 nitrogen setting, 50.0 psi; acquisition time: 10.0 min (Chinese Pharmacopoeia, 2012). The mass spectrum conditions of the four components agarotetrol, piperine, isoliquiritigenin, and ferulic acid are shown in Supplementary Table S2.

### Treatment methods and analysis conditions of tissue samples

#### Pre-treatment of samples

The tissue samples were accurately weighed and placed in a homogenizer, and pure water (0.02% formic acid) was added according to the mass-volume ratio of 1:2 to fully homogenize the samples. A 100-μl sample of uniform tissue homogenate was placed in a 2.0 ml Eppendorf tube (blank sample together with an equal volume of water or blank tissue homogenate), 900 μl of methanol (containing 0.1% formic acid) was added and the tube was vortexed for 5min, followed by ultrasonic mixing for 1 min. After centrifuging at 4°C for 5 min (12,000 rpm), 900 μl of the supernatant was transferred to a new test tube and dried in a 48°C water bath. A 100 μl equal proportion mobile phase was added to the precipitate before loading.

#### Analysis conditions

##### Chromatographic conditions

The following liquid chromatography system and conditions were used: Waters ACQUITY UPLC H-CLASS ultra-high performance liquid chromatography; column: Waters Acquity UPLC HSS T3 column (100 × 2.1 mm, 1.8 μm); MPA: 0.02% formic acid water; MPB: acetonitrile: methanol = 1:1 (containing 0.02% formic acid); flow rate: 0.300 ml min^−1^; column temperature: 40°C; sampling volume: 5.00 μl; the chromatographic gradients are shown in Supplementary Table S3 (Liu et al., 2020).

##### Mass spectrometry conditions

The following mass spectrometry (MS) conditions were used: MS/MS system: Q Exactive Orbitrap Mass Spectrometer; ion source: heated electrospray ion source; scan mode: full MS/dd-MS^2^ (Top 5); polarity: positive and negative ion scanning; resolution: 70000 FWHM (Full MS); 17500 FWHM (dd-MS^2^); scan range: 150–800 m·z^−1^; spray voltage: +3.8 kV/−2.8 kV; AGC target: 3e6 (Full MS); 1e5 (dd-MS^2^); microscans: 1; maximum inject time: 50.0 ms (Full MS); 50.0 ms (dd-MS^2^); isolation window: 2.00 m z^−1^; dynamic exclusion: 10.0 s; sheath gas flow rate: 35.0 arbs; auxiliary gas flow rate: 10.0 arbs; sweep gas flow rate: 0 arbs; capillary temperature: 325°C; heater temperature: 350°C; stepped normalized collision energy: 20.0, 40.0, and 60.0 eV (Liu et al., 2019).

### Data processing and analysis

The chromatogram collection and peak integration of analyte and internal standard were processed by MultiQuant 3.0.3 analysis software (AB Sciex). After optimizing the integration parameters, the target peak was automatically integrated and it was not allowed to integrate the peak separately or manually.

Calculation of the analyte concentration was determined by obtaining the chromatographic peak areas of the analyte and the internal standard using software Analyst 1.6.3. The ratio of the analyte concentration (x) to peak area (y) in plasma was linearly regressed using the weighted least squares method (W = 1/x^2^), and the regression equation (Y = a+bX) was the standard curve. The concentration of the analyte in the rat plasma was calculated according to the standard curve of the analysis batch, and the concentration unit was ng ml^−1^. The Phoenix WinNonlin non-compartment model module (version 8.1.0.3530) was used to calculate the pharmacokinetic parameters.

The statistical analysis assumed that the main pharmacokinetic parameters (C_max_ and AUC_0-t_) obeyed a normal distribution after logarithmic conversion. The LEVENE test was used to test the homogeneity of variance for the converted parameters. When the variance was uniform (*p* > 0.05), Student’s t-test was used to evaluate sex-based differences and single-factor variance analysis was used for dose difference. When the variance was uneven (*p* ≤ 0.05), the Mann–Whitney U-test (M–W method) was used for sex-based differences, while the Kruskal–Wallis H rank sum test was used to evaluate dose difference.

### Methodological verification

The results of the method verification are shown in Supplementary Figures S1–S6 and Supplementary Table S4 (EMEA, 1994; FDA, 2001; CFDA, 2014).

## Results

### Single administration

#### Drug–time curves of four components in plasma

**FIGURE 1:**
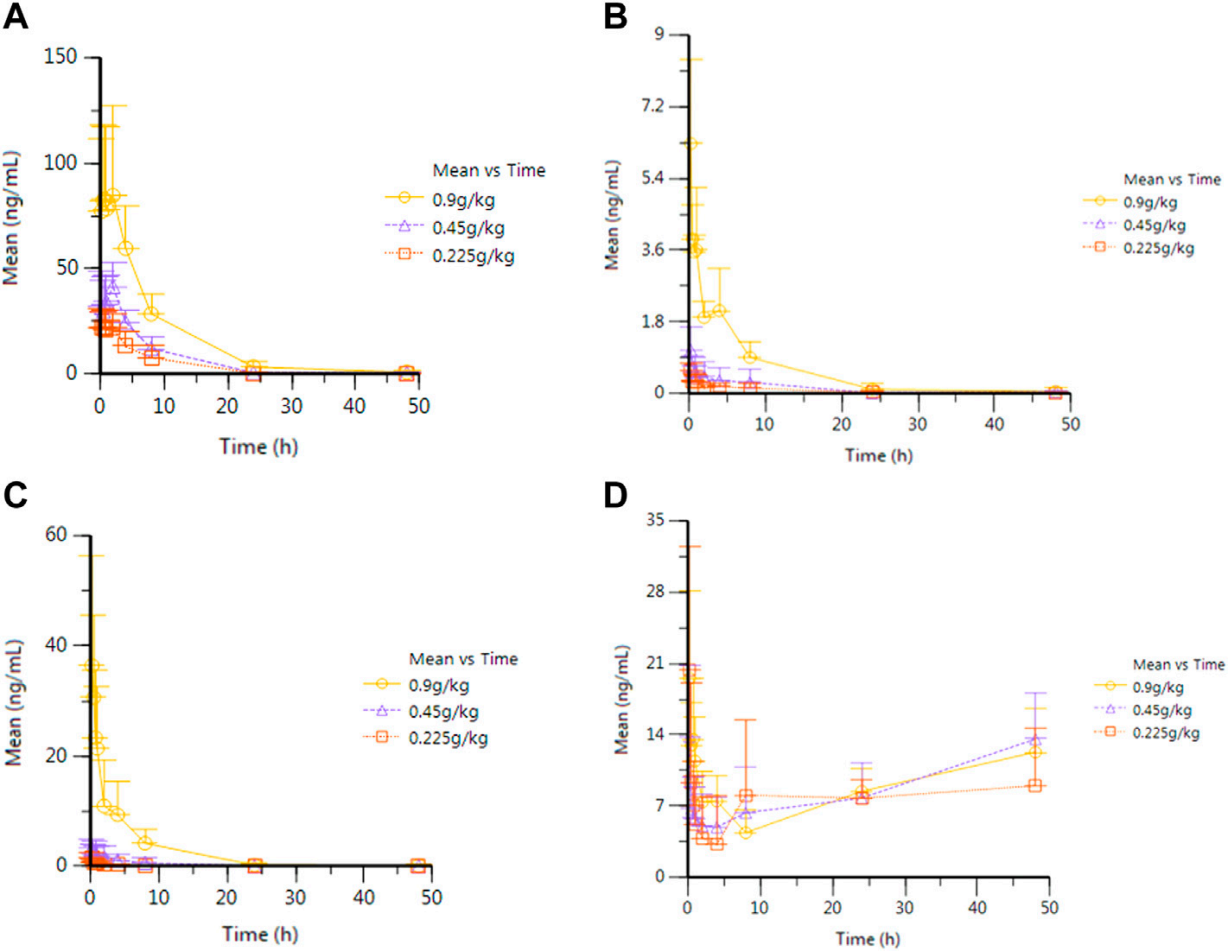
Average plasma concentration–time curve of eagleditol after different doses of RYZB. **(A)** Agarotetrol; **(B)** isoliquiritigenin; **(C)** piperine; **(D)** ferulic acid.

After oral administration of RYZB in rats, the mean plasma concentration–time curves of agarotetrol, isoliquiritigenin, piperine, and ferulic acid are shown in Figure 1.

#### Main pharmacokinetic parameters of the four components

After oral administration of RYZB in rats, the main pharmacokinetic parameters of agarotetrol, isoliquiritigenin, piperine, and ferulic acid are shown in Supplementary Tables S5–S8.

#### Investigation of linear pharmacokinetics in different dosages

**FIGURE 2:**
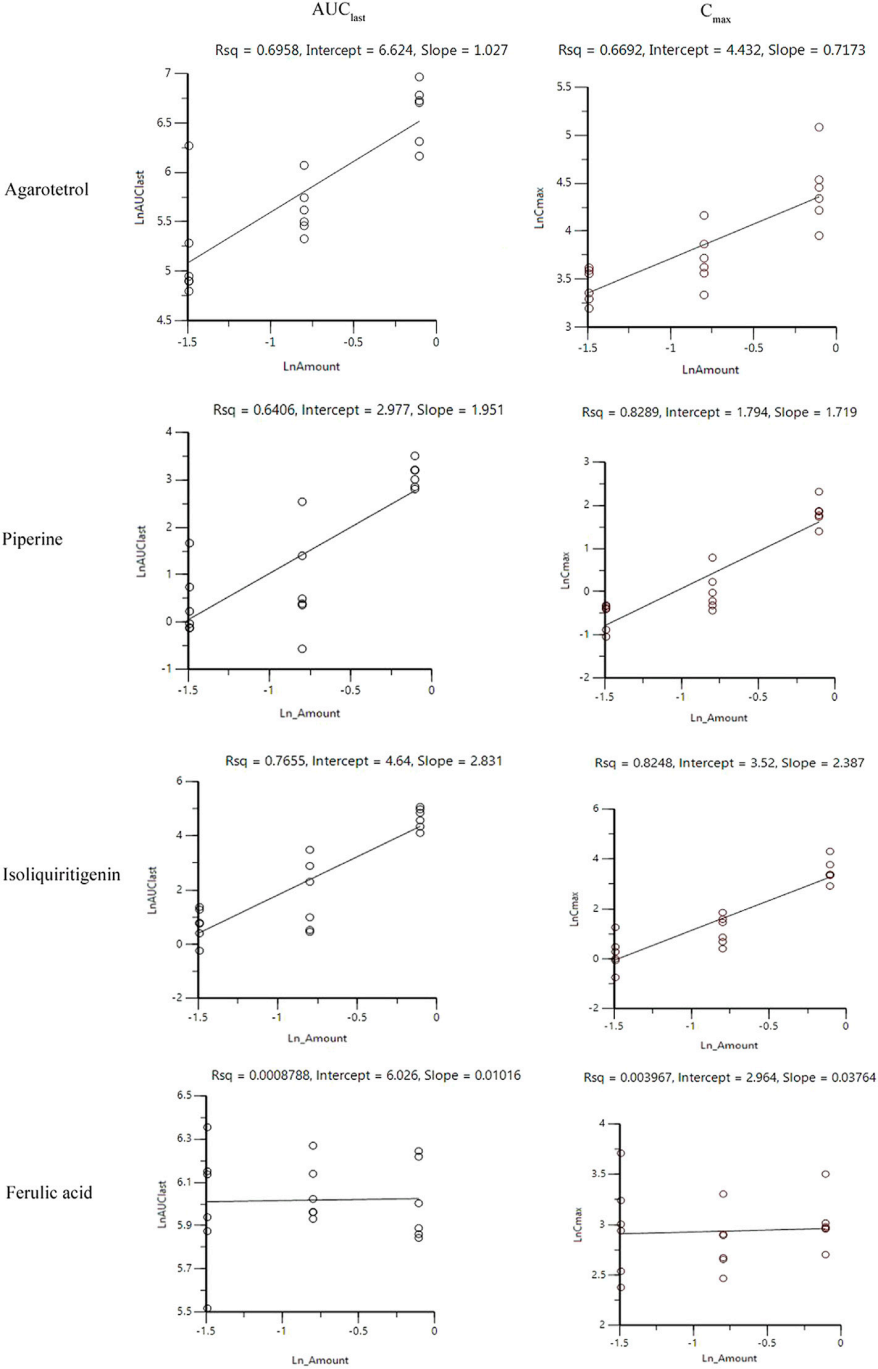
Linear relationship between the logarithmic dose of AUC_0-t_ and C_max_.

After oral administration of RYZB in the low, middle, and high groups in rats, the main pharmacokinetic parameters AUC_0-t_ and C_max_ were logarithmic and then linearly regressed with the logarithmic values of the doses given (see Figure 2 for details). The statistical results showed that the C_max_ and AUC_0-t_ of agarotetrol, isoliquiritigenin, and piperine changed significantly after the dose change (*p* < 0.01 or *p* < 0.001), and t_1/2_ and T_max_ were not affected by the dose change (*p* > 0.05) (see Supplementary Tables S5–S8 for details). However, the dose change had no effect on the C_max_, AUC_0-t_, and t_1/2_ of ferulic acid, but had a significant effect on the T_max_ (*p* < 0.05).

#### Comparison of differences in pharmacokinetic parameters caused by sex *in vivo*

The statistical results showed that sex-based differences have no influence on the main pharmacokinetic parameters of agarotetrol, isoliquiritigenin, piperine, and ferulic acid (*p* > 0.05) (see Supplementary Tables S5–S8 for details).

### Long-term administration

#### Plasma

##### Average drug–time curves of the four components in different administration cycles and different doses

**FIGURE 3:**
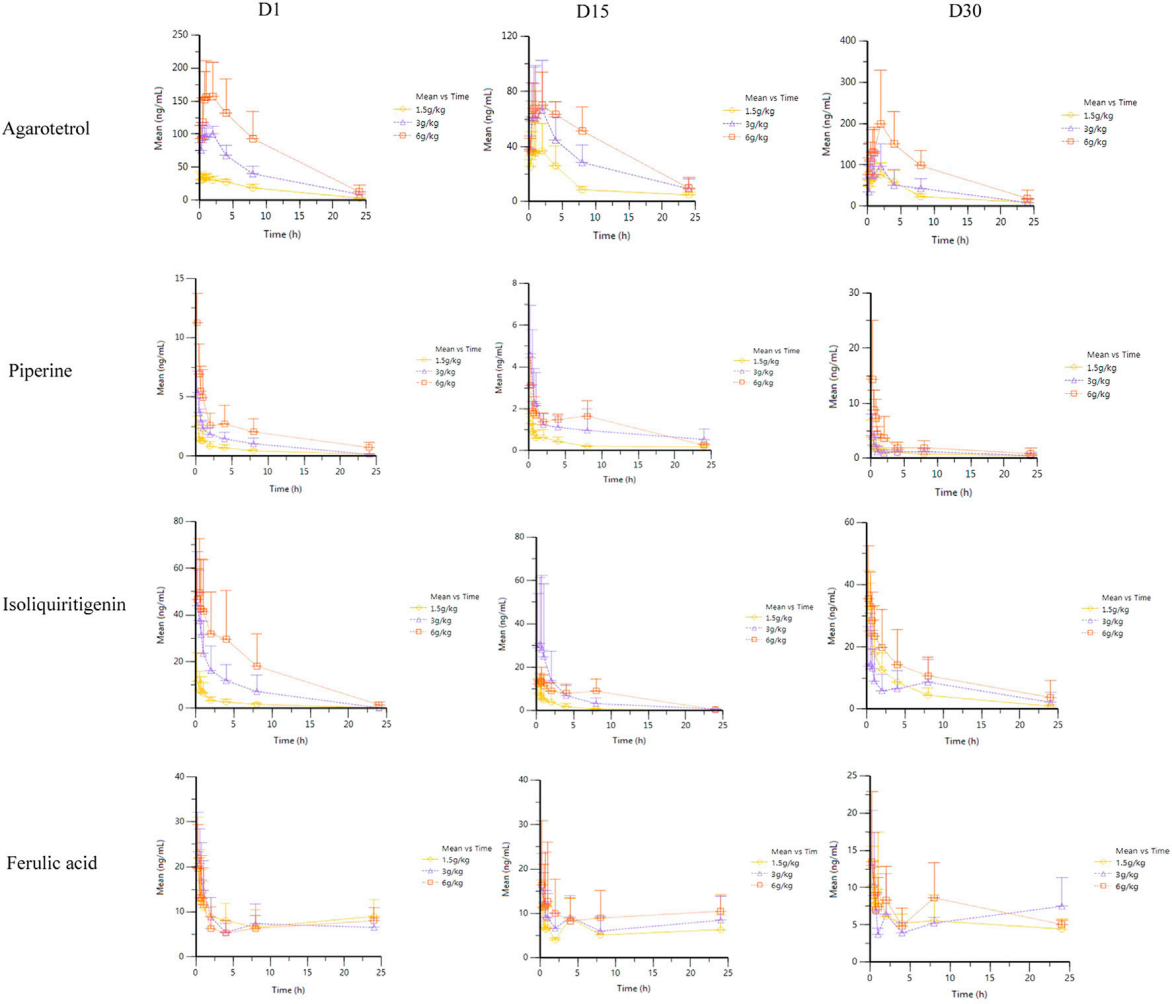
Average plasma concentration–time curve of four components at different times after different doses of RYZB.

Rats were given RYZB at a dose of 1.50, 3.00, or 6.00 g kg^−1^ orally. Average drug–time curves of agarotetrol, isoliquiritigenin, piperine, and ferulic acid in plasma at D1, D15, D30, and D15 of withdrawal are shown in Figure 3.

##### Main pharmacokinetic parameters

The pharmacokinetic parameters of agarotetrol, isoliquiritigenin, piperine, and ferulic acid at D1, D15, and D30 are given in Tables 1–Tables 12.

**TABLE 1:** Pharmacokinetic parameters of agarotetrol after different doses of TYZB in rats (D1).

Dose (g kg^−1^)	Sex	t_1/2_ (h)	T_max_ (h)	C_max_ (ng ml^−1^)	AUC_0-t_ (h ng ml^−1^)
1.5	M	6.03 ± 0.54	1.75 ± 1.50	31.83 ± 2.88	385.18 ± 73.80
	F	5.52 ± 1.17	1.31 ± 0.85	41.31 ± 10.42	327.03 ± 93.51
	Total	5.83 ± 0.76	1.53 ± 1.15	36.51 ± 8.72	356.36 ± 84.04
3.0	M	7.18 ± 1.86	1.31 ± 0.80	118.01 ± 16.91	937.17 ± 134.36
	F	6.56 ± 3.20	1.50 ± 0.58	101.33 ± 12.57	909.71 ± 121.35
	Total	6.97 ± 2.06	1.41 ± 0.65	109.34 ± 16.56	923.94 ± 119.07
6.0	M	7.08 ± 2.54	2.06 ± 1.53	169.17 ± 39.16	1,670.74 ± 628.58
	F	7.39 ± 6.62	1.06 ± 0.66	194.19 ± 56.84	2030.12 ± 559.67
	Total	7.26 ± 4.91	1.56 ± 1.22	182.74 ± 47.16	1850.20 ± 584.65
*p*	Dose	0.618	0.992	0.000***	0.000***
	Sex	0.203	0.590	0.820	0.980

Note: t_1/2_: elimination half-life; T_max_: time of maximum concentration; C_max_: maximum serum concentration; AUC_0-t_: area under serum drug concentration–time curve (from 0 h to t h). ***p < 0.001.

**TABLE 2:** Pharmacokinetic parameters of agarotetrol after different doses of TYZB in rats (D15).

Dose (g kg^−1^)	Sex	t_1/2_ (h)	T_max_ (h)	C_max_ (ng ml^−1^)	AUC_0-t_ (h ng ml^−1^)
1.5	M	13.24 ± 12.30	1.31 ± 0.80	30.81 ± 8.26	212.01 ± 54.75
	F	4.64 ± 9.85	3.25 ± 1.50	51.04 ± 28.16	369.15 ± 81.14
	Total	11.51 ± 11.35	2.28 ± 1.52	40.90 ± 22.08	291.18 ± 106.36
3.0	M	6.87 ± 2.82	1.63 ± 0.75	56.00 ± 19.23	540.42 ± 95.50
	F	15.84 ± 13.05	1.44 ± 0.66	87.24 ± 47.38	785.44 ± 328.15
	Total	11.40 ± 9.96	1.53 ± 0.66	71.61 ± 37.35	662.79 ± 259.17
6.0	M	4.02 ± 0.24	2.19 ± 1.34	61.82 ± 5.52	753.26 ± 156.00
	F	7.00 ± 2.66	2.88 ± 3.47	91.44 ± 18.15	1,120.44 ± 206.78
	Total	5.51 ± 2.35	2.53 ± 2.46	76.65 ± 20.10	935.58 ± 258.63
*p*	Dose	0.262	0.716	0.009**	0.000***
	Sex	0.310	0.443	0.060	0.080

Note: t_1/2_: elimination half-life; T_max_: time of maximum concentration; C_max_: maximum serum concentration; AUC_0-t_: area under serum drug concentration–time curve (from 0 h to t h). ***p < 0.001.

**TABLE 3:** Pharmacokinetic parameters of agarotetrol after different doses of TYZB in rats (D30).

Dose (g kg^−1^)	Sex	t_1/2_ (h)	T_max_ (h)	C_max_ (ng ml^−1^)	AUC_0-t_ (h ng ml^−1^)
1.5	M	3.66 ± 0.41	1.75 ± 0.500	54.92 ± 11.01	464.11 ± 63.40
	F	5.13 ± 1.68	2.00 ± 1.41	112.74 ± 11.95	817.55 ± 151.47
	Total	4.29 ± 1.28	1.88 ± 0.991	83.35 ± 32.21	641.45 ± 217.52
3.0	M	22.01 ± 15.4774	1.56 ± 1.63	16.08 ± 18.81	28.41 ± 8.14
	F	6.02 ± 3.55	1.69 ± 0.625	125.25 ± 14.46	1,130.01 ± 72.95
	Total	12.92 ± 12.67	1.63 ± 1.14	70.67 ± 60.43	578.24 ± 589.54
6.0	M	10.82 ± 13.00	1.44 ± 0.657	193.15 ± 177.38	1,630.07 ± 836.21
	F	8.17 ± 5.24	2.50 ± 1.00	220.58 ± 59.34	2,450.52 ± 418.29
	Total	9.69 ± 9.76	1.97 ± 0.968	207.31 ± 123.72	2040.14 ± 751.44
*p*	Dose	0.086	0.470	0.007**	0.002**
	Sex	0.756	0.198	0.005**	0.007**

Note: t_1/2_: elimination half-life; T_max_: time of maximum concentration; C_max_: maximum serum concentration; AUC_0-t_: area under serum drug concentration–time curve (from 0 h to t h). **p < 0.01.

**TABLE 4:** Pharmacokinetic parameters of isoliquiritigenin after different doses of TYZB in rats (D1).

Dose (g kg^−1^)	Sex	t_1/2_ (h)	T_max_ (h)	C_max_ (ng ml^−1^)	AUC_0-t_ (h ng ml^−1^)
1.5	M	9.54 ± 8.26	0.25 ± 0.00	2.18 ± 0.411	5.62 ± 0.87
	F	6.80 ± 2.46	0.44 ± 0.38	3.04 ± 0.99	9.29 ± 4.35
	Total	8.17 ± 5.83	0.34 ± 0.27	2.61 ± 0.84	7.45 ± 3.50
3.0	M	5.12 ± 1.03	0.31 ± 0.13	4.06 ± 0.50	13.21 ± 4.00
	F	5.86 ± 1.23	0.25 ± 0.00	6.83 ± 1.38	20.94 ± 10.10
	Total	5.49 ± 1.12	0.28 ± 0.09	5.44 ± 1.77	17.07 ± 8.21
6.0	M	10.71 ± 2.01	0.31 ± 0.13	13.54 ± 0.96	47.34 ± 12.70
	F	23.91 ± 33.42	0.25 ± 0.00	9.78 ± 1.76	33.57 ± 16.48
	Total	17.30 ± 23.04	0.28 ± 0.09	11.60 ± 2.37	40.44 ± 15.56
*p*	Dose	0.0680	0.994	0.000***	0.000***
	Sex	0.843	0.799	0.570	0.680

Note: t_1/2_: elimination half-life; T_max_: time of maximum concentration; C_max_: maximum serum concentration; AUC_0-t_: area under serum drug concentration–time curve (from 0 h to t h). ***p < 0.001.

**TABLE 5:** Pharmacokinetic parameters of isoliquiritigenin after different doses of TYZB in rats (D15).

Dose (g kg^−1^)	Sex	t_1/2_ (h)	T_max_ (h)	C_max_ (ng ml^−1^)	AUC_0-t_ (h ng ml^−1^)
1.5	M	6.24 ± 3.45	0.25 ± 0.00	0.93 ± 0.27	3.94 ± 1.97
	F	6.84 ± 4.96	0.31 ± 0.13	1.83 ± 0.51	4.95 ± 2.20
	Total	6.54 ± 3.97	0.28 ± 0.09	1.38 ± 0.61	4.45 ± 2.01
3.0	M	7.80 ± 5.02	0.38 ± 0.25	3.12 ± 1.98	11.96 ± 3.14
	F	42.84 ± 59.81	0.31 ± 0.13	6.34 ± 1.87	21.77 ± 4.13
	Total	25.34 ± 43.53	0.34 ± 0.19	4.73 ± 2.48	16.80 ± 6.28
6.0	M	16.22 ± 9.02	0.50 ± 0.35	2.67 ± 0.99	15.11 ± 6.74
	F	15.24 ± 11.85	0.25 ± 0.00	3.70 ± 1.37	20.46 ± 16.94
	Total	15.77 ± 9.41	0.38 ± 0.27	3.19 ± 1.24	17.75 ± 12.24
*p*	Dose	0.056	0.738	0.000***	0.000***
	Sex	0.365	0.671	0.030*	0.290

Note: t_1/2_: elimination half-life; T_max_: time of maximum concentration; C_max_: maximum serum concentration; AUC_0-t_: area under serum drug concentration–time curve (from 0 h to t h). ***p < 0.001; *p < 0.05.

**TABLE 6:** Pharmacokinetic parameters of isoliquiritigenin after different doses of TYZB in rats (D30).

Dose (g kg^−1^)	Sex	t_1/2_ (h)	T_max_ (h)	C_max_ (ng ml^−1^)	AUC_0-t_ (h ng ml^−1^)
1.5	M	7.67 ± 4.90	2.25 ± 3.84	1.80 ± 0.62	7.15 ± 1.93
	F	9.57 ± 5.45	0.31 ± 0.13	5.93 ± 3.20	16.77 ± 6.70
	Total	8.75 ± 4.89	1.28 ± 2.72	3.87 ± 3.07	11.90 ± 6.85
3.0	M	1.60 ± 0.97	0.69 ± 0.13	1.27 ± 1.15	1.44 ± 1.49
	F	11.80 ± 6.66	0.25 ± 0.00	6.91 ± 2.25	20.08 ± 6.82
	Total	9.72 ± 7.34	0.47 ± 0.25	4.09 ± 3.44	10.74 ± 10.90
6.0	M	14.42 ± 16.82	0.81 ± 0.83	14.74 ± 3.45	41.68 ± 18.45
	F	12.90 ± 7.40	0.25 ± 0.00	15.57 ± 15.46	53.21 ± 14.04
	Total	13.74 ± 12.04	0.53 ± 0.62	15.15 ± 10.30	47.84 ± 16.38
*p*	Dose	0.856	0.733	0.003**	0.000***
	Sex	0.157	0.008**	0.119	0.030*

Note: t_1/2_: elimination half-life; T_max_: time of maximum concentration; C_max_: maximum serum concentration; AUC_0-t_: area under serum drug concentration–time curve (from 0 h to t h). ***p < 0.001; **p < 0.01; *p < 0.05.

**TABLE 7:** Pharmacokinetic parameters of piperine after different doses of TYZB in rats (D1).

Dose (g kg^−1^)	Sex	t_1/2_ (h)	T_max_ (h)	C_max_ (ng ml^−1^)	AUC_0-t_ (h ng ml^−1^)
1.5	M	4.65 ± 0.51	0.63 ± 0.43	6.50 ± 2.38	18.81 ± 4.66
	F	4.42 ± 0.14	0.44 ± 0.38	17.49 ± 16.16	47.32 ± 9.39
	Total	4.52 ± 0.33	0.53 ± 0.39	11.94 ± 12.18	33.02 ± 16.72
3.0	M	3.77 ± 1.87	0.38 ± 0.25	25.26 ± 6.63	72.32 ± 43.82
	F	3.13 ± 0.85	0.25 ± 0.00	64.17 ± 10.29	171.82 ± 43.75
	Total	3.45 ± 1.39	0.31 ± 0.18	44.60 ± 22.31	122.21 ± 66.62
6.0	M	8.95 ± 9.75	0.50 ± 0.20	52.74 ± 22.87	392.15 ± 225.55
	F	11.64 ± 11.56	1.25 ± 1.84	53.40 ± 17.55	301.18 ± 145.19
	Total	10.19 ± 9.67	0.87 ± 1.27	53.04 ± 18.87	347.20 ± 182.36
*p*	Dose	0.047*	0.197	0.000***	0.000***
	Sex	1.000	0.410	0.120	0.250

Note: t_1/2_: elimination half-life; T_max_: time of maximum concentration; C_max_: maximum serum concentration; AUC_0-t_: area under serum drug concentration-time curve (from 0 h to t h). ***p < 0.001; *p < 0.05.

**TABLE 8:** Pharmacokinetic parameters of piperine after different doses of TYZB in rats (D15).

Dose (g kg^−1^)	Sex	t_1/2_ (h)	T_max_ (h)	C_max_ (ng ml^−1^)	AUC_0-t_ (h ng ml^−1^)
1.5	M	4.23 ± 1.64	0.25 ± 0.00	4.64 ± 1.72	14.18 ± 10.21
	F	3.59 ± 2.91	0.31 ± 0.13	13.17 ± 10.24	28.88 ± 24.35
	Total	3.91 ± 2.21	0.28 ± 0.09	8.86 ± 8.16	21.41 ± 19.00
3.0	M	11.63 ± 16.42	0.31 ± 0.125	19.44 ± 10.46	62.25 ± 24.57
	F	2.53 ± 1.32	0.50 ± 0.29	48.97 ± 43.38	132.31 ± 117.55
	Total	7.06 ± 11.87	0.41 ± 0.23	34.20 ± 33.12	97.34 ± 86.95
6.0	M	6.05 ± 1.79	0.87 ± 0.78	15.07 ± 3.30	85.85 ± 45.45
	F	5.24 ± 1.58	2.38 ± 3.76	19.37 ± 6.32	144.54 ± 113.37
	Total	5.78 ± 1.61	1.63 ± 2.64	17.24 ± 5.19	115.57 ± 85.64
*p*	Dose	0.103	0.027*	0.002**	0.001**
	Sex	0.123	0.443	0.060	0.320

Note: t_1/2_: elimination half-life; T_max_: time of maximum concentration; C_max_: maximum serum concentration; AUC_0-t_: area under serum drug concentration–time curve (from 0 h to t h). **p < 0.01; *p < 0.05.

**TABLE 9:** Pharmacokinetic parameters of piperine after different doses of TYZB in rats (D30).

Dose (g kg^−1^)	Sex	t_1/2_ (h)	T_max_ (h)	C_max_ (ng ml^−1^)	AUC_0-t_ (h ng ml^−1^)
1.5	M	3.47 ± 1.16	1.19 ± 0.94	12.84 ± 5.97	63.93 ± 39.11
	F	6.38 ± 6.39	0.31 ± 0.13	41.67 ± 13.61	130.47 ± 27.90
	Total	4.93 ± 4.52	0.75 ± 0.78	27.22 ± 18.24	97.14 ± 47.46
3.0	M	2.86 ± 1.40	0.69 ± 0.13	2.43 ± 2.08	1.42 ± 0.90
	F	10.91 ± 9.30	0.37 ± 0.14	31.24 ± 6.06	159.25 ± 76.84
	Total	8.24 ± 8.35	0.53 ± 0.21	16.85 ± 15.94	80.14 ± 98.05
6.0	M	12.14 ± 17.17	1.00 ± 0.74	32.50 ± 12.99	195.25 ± 129.51
	F	11.31 ± 8.50	0.25 ± 0.00	47.63 ± 8.80	314.36 ± 51.74
	Total	11.75 ± 12.51	0.63 ± 0.63	40.04 ± 13.05	255.01 ± 111.35
*p*	Dose	0.381	0.809	0.041*	0.009**
	Sex	0.156	0.001**	0.001*	0.011

Note: t_1/2_: elimination half-life; T_max_: time of maximum concentration; C_max_: maximum serum concentration; AUC_0-t_: area under serum drug concentration–time curve (from 0 h to t h). **p < 0.01; *p < 0.05.

**TABLE 10:** Pharmacokinetic parameters of ferulic acid after different doses of TYZB in rats (D1).

Dose (g kg^−1^)	Sex	t_1/2_ (h)	T_max_ (h)	C_max_ (ng ml^−1^)	AUC_0-t_ (h ng ml^−1^)
1.5	M	193.02 ± 231.15	0.25 ± 0.00	17.01 ± 6.18	179.38 ± 58.61
	F	53.64 ± 33.37	0.25 ± 0.00	26.91 ± 9.33	209.24 ± 74.97
	Total	100.17 ± 128.52	0.25 ± 0.00	21.94 ± 9.05	194.21 ± 64.25
3.0	M	51.07 ± 59.08	0.37 ± 0.14	20.84 ± 6.24	146.20 ± 12.96
	F	17.77 ± 15.44	0.25 ± 0.00	26.80 ± 10.11	179.25 ± 98.95
	Total	32.07 ± 40.05	0.31 ± 0.12	23.83 ± 8.39	162.24 ± 67.75
6.0	M	18.51 ± 26.45	0.31 ± 0.12	13.24 ± 3.46	89.64 ± 86.65
	F	12.47 ± 10.13	0.44 ± 0.38	27.54 ± 7.85	141.37 ± 84.58
	Total	15.58 ± 18.29	0.38 ± 0.27	20.30 ± 9.48	115.24 ± 83.87
*p*	Dose	0.064	0.317	0.636	0.071
	Sex	0.657	0.551	0.002**	0.450

Note: t_1/2_: elimination half-life; T_max_: time of maximum concentration; C_max_: maximum serum concentration; AUC_0-t_:area under serum drug concentration–time curve (from 0 h to t h). **p < 0.01.

**TABLE 11:** Pharmacokinetic parameters of ferulic acid after different doses of TYZB in rats (D15).

Dose (g kg^−1^)	Sex	t_1/2_ (h)	T_max_ (h)	C_max_ (ng ml^−1^)	AUC_0-t_ (h ng ml^−1^)
1.5	M	15.17 ± 13.50	6.19 ± 11.93	7.32 ± 1.78	74.74 ± 50.15
	F	13.25 ± 13.17	6.25 ± 11.80	16.61 ± 5.09	118.36 ± 94.86
	Total	14.05 ± 12.18	6.22 ± 11.01	11.94 ± 6.07	96.24 ± 73.85
3.0	M	19.16 ± 2.55	1.25 ± 1.84	18.04 ± 9.00	132.35 ± 30.64
	F	18.17 ± 13.38	0.31 ± 0.12	15.37 ± 7.19	171.58 ± 146.69
	Total	18.57 ± 9.51	0.78 ± 1.31	16.73 ± 7.68	152.22 ± 99.61
6.0	M	16.85 ± 3.47	12.12 ± 13.77	9.85 ± 2.55	155.74 ± 81.35
	F	15.34 ± 15.23	2.38 ± 3.77	26.54 ± 16.20	287.25 ± 88.14
	Total	15.97 ± 10.92	7.25 ± 10.7	18.21 ± 14.05	221.52 ± 106.74
*p*	Dose	0.778	0.621	0.389	0.126
	Sex	0.464	0.799	0.020*	0.580

Note: t_1/2_: elimination half-life; T_max_: time of maximum concentration; C_max_: maximum serum concentration; AUC_0-t_: area under serum drug concentration–time curve (from 0 h to t h). *p < 0.05.

**TABLE 12:** Pharmacokinetic parameters of ferulic acid after different doses of TYZB in rats (D30).

Dose (g kg^−1^)	Sex	t_1/2_ (h)	T_max_ (h)	C_max_ (ng ml^−1^)	AUC_0-t_ (h ng ml^−1^)
1.5	M	12.51 ± 20.74	0.31 ± 0.13	7.70 ± 5.73	2.96 ± 2.12
	F	14.57 ± 11.54	0.56 ± 0.38	15.42 ± 4.77	103.21 ± 74.58
	Total	13.56 ± 15.08	0.44 ± 0.29	11.55 ± 6.38	52.71 ± 56.98
3.0	M	14.54 ± 1.80	8.75 ± 13.22	6.78 ± 3.07	88.02 ± 75.24
	F	18.75 ± 14.78	6.19 ± 11.91	17.85 ± 4.02	145.21 ± 34.64
	Total	17.35 ± 11.78	7.29 ± 11.44	7.29 ± 11.45	13.14 ± 6.80
6.0	M	24.62 ± 8.70	2.63 ± 3.58	9.04 ± 2.93	58.67 ± 68.68
	F	19.32 ± 11.95	2.65 ± 3.98	21.04 ± 7.99	116.25 ± 61.75
	Total	21.14 ± 10.45	2.64 ± 3.40	15.07 ± 8.48	87.51 ± 67.84
*p*	Dose	0.431	0.117	0.610	0.180
	Sex	0.751	0.481	0.000***	0.003**

Note: t_1/2_: elimination half-life; T_max_: time of maximum concentration; C_max_: maximum serum concentration; AUC_0-t_: area under serum drug concentration–time curve (from 0 h to t h). ***p < 0.001; **p < 0.01.

##### Investigation of linear pharmacokinetics for different dosages

**FIGURE 4:**
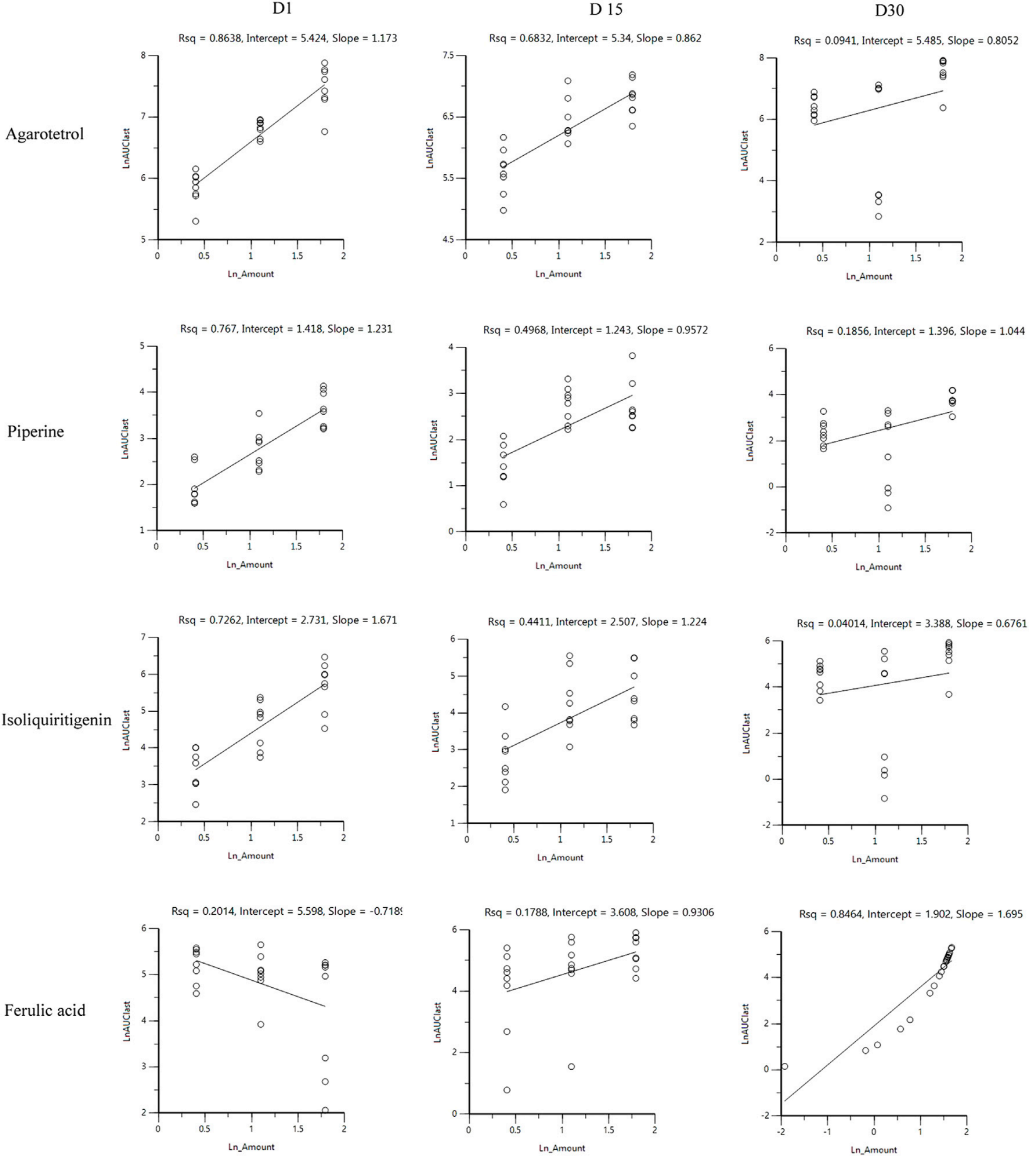
Linear relationship diagram between the logarithmic dose value and the logarithmic AUC_0-t_ value.

**FIGURE 5:**
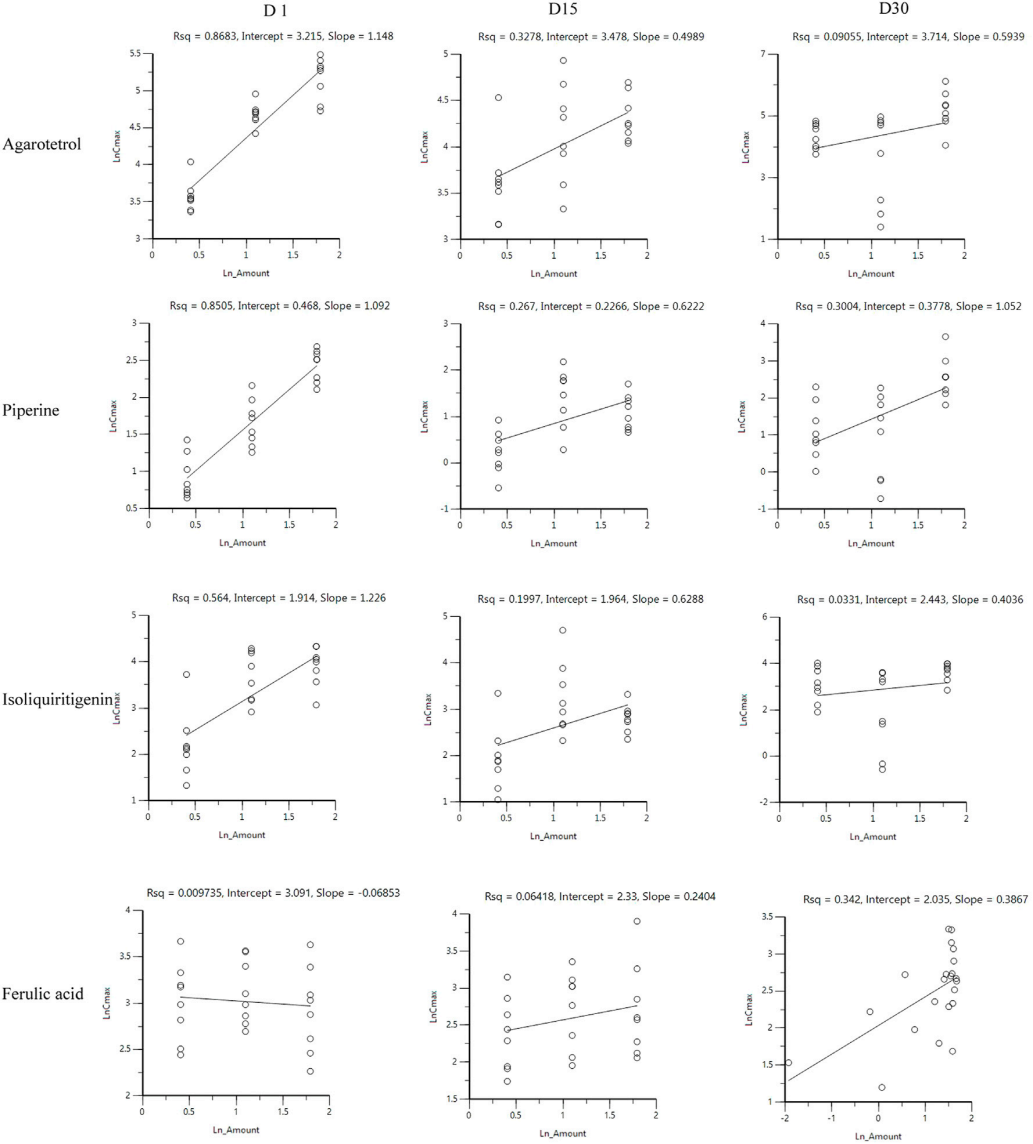
Linear relationship diagram between the logarithmic dose value and the logarithmic C_max_ value.

After oral administration of RYZB in the low, middle, and high dose groups in rats, the main pharmacokinetic parameters AUC_0-t_ and C_max_ increased logarithmically and then linearly regressed with the logarithmic values of the given doses, as shown in Figures 4, 5.

On D1 after administration, C_max_ and AUC_0-t_ of agarotetrol and isoliquiritigenin changed significantly (*p* < 0.05), and t_1/2_ and T_max_ were not affected by dose change (*p* > 0.05). C_max_, AUC_0-t_, and t_1/2_ of piperine changed significantly (*p* < 0.05), but did not have a significant effect on the T_max_ (*p* > 0.05). There was no significant effect on C_max_, AUC_0-t_, t_1/2_, or the T_max_ of ferulic acid (*p* > 0.05); details are given in Tables 1, 4, 7, 10.

On D15 after administration, C_max_ and AUC_0-t_ of agarotetrol and isoliquiritigenin changed significantly (*p* < 0.05), although t_1/2_ and T_max_ were not affected by dose changes (*p* > 0.05); C_max_, AUC_0-t_, and T_max_ of piperine changed significantly (*p* < 0.05), but had no significant effect on t_1/2_ (*p* > 0.05). The dosage change had no significant effects on C_max_, AUC_0-t_, T_max_, or t_1/2_ of ferulic acid (*p* > 0.05), details are given in Tables 2, 5, 8, 11.

On D30 after administration, C_max_ and AUC_0-t_ of agarotetrol, isoliquiritigenin, and piperine changed significantly (*p* < 0.05), but had no significant effect on t_1/2_ or T_max_ (*p* > 0.05). The dosage change had no significant effect on C_max_, AUC_0-t_, T_max_, or the t_1/2_ of ferulic acid (*p* > 0.05), details are given in Tables 3, 6, 9, 12.

##### Comparison of sex-based differences in pharmacokinetic parameters in vivo

Agaroterol: on D1 and D15 of administration, sex did not have an effect on the main pharmacokinetic parameters of agaroterol (*p* > 0.05). On D30 of administration, sex-based differences were significant, with differences in C_max_ and AUC_0-t_ (*p* < 0.05), while t_1/2_ and T_max_ were not affected by sex (*p* > 0.05); see Tables 1–Tables 3 for details.

Isoliquiritigenin: the pharmacokinetic parameters of isoliquiritigenin were not affected by sex on D1 of administration (*p* > 0.05). After D15 of administration, sex had a significant effect on C_max_ (*p* < 0.05), but had no effect on other pharmacokinetic parameters (*p* > 0.05). On the D30 of administration, sex had a significant effect on AUC_0-t_ and T_max_ (*p* < 0.05), but had no effect on C_max_ and t_1/2_ (*p* > 0.05); see Tables 4–Tables 6 for details.

Piperine: on D1 and D15 of administration, the results showed that sex had no effect on the pharmacokinetic parameters of piperine (*p* > 0.05). After D30 of administration, sex significantly influenced the pharmacokinetic parameters C_max_, AUC_0-t_, and T_max_ (*p* < 0.05), but not t_1/2_ (*p* > 0.05); see Tables 7–Tables 9 for details.

Ferulic acid: on D1 and D15 of administration, sex had significant influence on C_max_ (*p* < 0.05), but had no influence on other pharmacokinetic parameters (*p* > 0.05). On D30 of administration, the results showed that sex had significant effects on AUC_0-t_ and C_max_ (*p* < 0.05), but had no significant effects on t_1/2_ or T_max_ (*p* > 0.05); see Tables 10–Tables12 for details.

##### Test results of the four components after 15 days of withdrawal

After 15 days of withdrawal, agarotetrol and piperine were not detected for any of the three doses; isoliquiritigenin and ferulic acid could be detected in the low dose group, while ferulic acid could be detected in the middle and high doses; see Table 13 for details.

**TABLE 13:** Test results of four components after 15 days of withdrawal.

Ingredient	Concentration (ng ml^−1^)		
	1.5 (g kg^−1^)	3.0 (g kg^−1^)	6.0 (g kg^−1^)
Agarotetrol	BQL	BQL	BQL
Isoliquiritigenin	0.23 ± 0.14	BQL	BQL
Piperine	BQL	BQL	BQL
Ferulic acid	5.45	2.88	4.45 ± 1.34

##### Accumulation index of systemic exposure of four components in plasma

The average ratios of AUC_0-t_ for the low, middle, and high dose groups of agarotetrol in plasma were 2.39, 0.77, and 1.10, respectively, and the C_max_ ratio was 2.28, 0.65, and 1.14, respectively. The average ratios of AUC_0-t_ and C_max_ for isoliquiritigenin were 1.60, 0.63, and 1.17, respectively, and 1.48, 0.75, and 1.30 respectively. The average ratios of AUC_0-t_ and C_max_ for piperine were 2.94, 0.657, and 0.735, respectively, and 2.29, 0.38, and 0.76, respectively. The average ratios of AUC_0-t_ and C_max_ for ferulic acid were 0.27, 0.08, and 0.76, and 0.53, 0.31, and 0.74, respectively; see Table 14 for details.

**TABLE 14:** Accumulation index of systemic exposure of four components in plasma (ratio).

	Dose (g kg^−1^)	Agarotetrol		Isoliquiritigenin		Piperine		Ferulic acid	
		AUC_0-t_	C_max_	AUC_0-t_	C_max_	AUC_0-t_	C_max_	AUC_0-t_	C_max_
Female	1.5	2.49	2.71	1.80	1.95	2.75	2.39	0.49	0.57
	3.0	1.24	1.24	0.96	1.01	0.93	0.49	0.81	0.66
	6.0	1.21	1.13	1.59	1.58	1.04	0.89	0.82	0.76
Male	1.5	1.21	1.73	1.27	0.83	3.40	1.97	0.02	0.45
	3.0	0.03	0.14	0.11	0.31	0.02	0.09	0.60	0.33
	6.0	0.98	1.13	0.88	1.09	0.50	0.62	0.65	0.69
Total	1.5	2.39	2.28	1.60	1.48	2.94	2.29	0.27	0.53
	3.0	0.77	0.65	0.63	0.75	0.66	0.38	0.08	0.31
	6.0	1.10	1.14	1.17	1.30	0.74	0.76	0.76	0.74

#### Tissue

##### Contents of four components in different tissues at different doses

**FIGURE 6:**
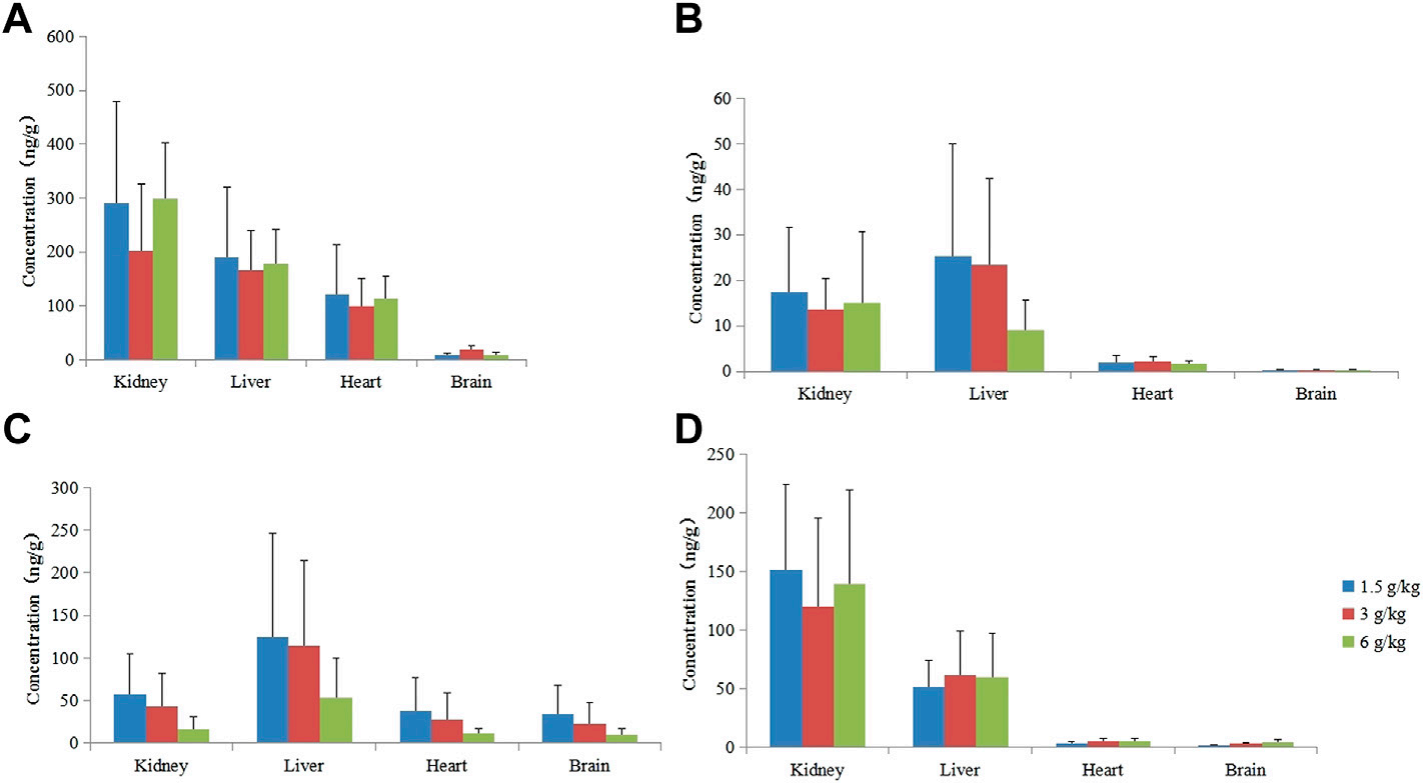
Concentration of four components in different tissues at different doses. **(A)** Agarotetrol; **(B)** isoliquiritigenin; **(C)** piperine; **(D)** ferulic acid.

After rats were given 1.50, 3.00, and 6.00 g kg^−1^ of RYZB, agarotetrol, ferulic acid, piperine, and isoliquiritigenin were detected in the brain, heart, liver, and kidney tissues and their concentrations were higher in the kidney and liver, but lower in the heart and brain tissues. Agarotetrol was distributed mainly to the kidney, liver, and heart, but less to the brain. Isoliquiritigenin was distributed more to the kidney and liver, but less to the brain and heart. Piperine was distributed more in the liver and distributed more to the brain than the other three substances, and was less distributed to the heart and kidney. Ferulic acid was mainly distributed to the kidney, followed by the liver, but was less distributed to the brain and heart; see Figure 6 for details.

##### Qualitative analysis of tissue samples

After RYZB was administered to rats, tissue samples were analyzed and processed using a combination of full MS/dd-MS2 and the database mzCloud and ChemSpider. Fourteen components were identified in brain samples, which were 7-hydroxy-3′,4′-dimethoxyisofavone, 2′-methoxyformononetin, vestitone, sativanone, piperine, piperlongumine, pipernonaline, tryptophan, liquiritin, isocostic acid, glycocholic acid, lecithos, oleic acid, and cholan-24-oic acid. A total of 18 components were detected in the heart: agarotetrol, linolenic acid, 7-hydroxy-3′,4′-dimethoxyisoflavone, 2′-methoxyformononetin, daidzin, vestitone, sativanone, piperine, piperlongumine, tryptophan, liquiritin, isocostic acid, linoleic acid, glycocholic acid, glycochenodeoxycholic acid, corchoionoside C, oleic acid, and cholan-24-oic acid. There were 24 components detected in the liver: agarotetrol, linolenic acid, 7-hydroxy-3′,4′- dimethoxyisoflavone, 2-methoxyformononetin, daidzein, isoliquiritigenin, vestitone, sativanone, piperine, piperlongumine, pipernonaline, apigenin, tryptophan, liquiritin, ferulic acid, isocostic acid, linoleic acid, normuscone, glycocholic acid, glycochenodeoxycholic acid, lecithos, corchoionoside C, oleic acid, and cholan-24-oic acid. A total of 24 components were detected in the kidney: agarotetrol, linolenic acid, 7-hydroxy-3′,4′-dimethoxyisoflavone, 2′-methoxyformononetin, daidzein, isoliquiritigenin, vestitone, sativanone, piperine, piperlongumine, pipernonaline, apigenin, tryptophan, liquiritin, ferulic acid, isocostic acid, dehydrocostus lactone, linoleic acid, glycocholic acid, glycochenodeoxycholic acid, lecithos, corchoionoside C, oleic acid, and cholan-24-oic acid.

## Discussion

Hydroxysafflor yellow A was the quality control index of the Ruyi Zhenbao tablet, but in the early blood components, hydroxysafflor yellow A was not detected, which was closely related to its low bioavailability. According to literature reports, hydroxysafflor yellow A was poorly absorbed by rats, and its absolute bioavailability is 1. 2%, which has a bile efflux effect (Li et al., 2015; Chen et al., 2022). So the other four components were chosen to be detected. Through the pharmacokinetic study of the bioactive components of RYZB, such as agaroterol, piperine, isoliquiritigenin, and ferulic acid, we could understand their changing rules in the body and provide a reasonable basis for clinical applications.

After a single administration, the T_max_ of agarotetrol, isoliquiritigenin, piperine, and ferulic acid in plasma was achieved mainly between 0.25 and 4 h. The C_max_ of the high dose was 89.5 ± 37.9 ng ml^−1^, 6.42 ± 2.02 ng ml^−1^, 36.8 ± 19.6 ng ml^−1^, and 21.1 ± 6.22 ng ml^−1^, respectively. AUC_0-t_ was 768 ± 217 h ng ml^−1^, 22.8 ± 6.16 h ng ml^−1^, 110 ± 38.8 h ng ml^−1^, and 413 ± 76.9 h ng ml^−1^, respectively. The plasma t_1/2_ was 6.84 ± 2.40 h, 8.65 ± 5.42 h, 5.34 ± 3.44 h, and 82.6 ± 42.7 h respectively. MRT_0-t_ was 6.69 ± 1.82 h, 5.63 ± 3.52 h, 4.33 ± 1.96 h, and 28.0 ± 3.74 h, respectively.

The T-test and the rank sum test were used to evaluate the dose differences of the main pharmacokinetic parameters. The results showed that the dose change had no significant effect on t_1/2_ and T_max_ of agarotetrol, isoliquiritigenin, and piperine (*p* > 0.05). The aforementioned results showed that in the dose range of 0.225–0.900 g kg^−1^, the increase in the AUC_0-t_ and C_max_ values of agarotetrol, isoliquiritigenin, and piperine was related to the dose increase. There were no significant differences in C_max_ and AUC_0-t_ of ferulic acid at different doses (*p* > 0.05), mainly because ferulic acid was not only an effective component of TCM but also a functional phenolic acid component in plant cell walls, which was commonly found in grains and nuts (Russell and Duthie, 2011; Tinikul et al., 2018; Kumar and Goel, 2019). After 4 h of administration, the rats were fed again and, thus, there was no significant difference in the low, middle, and high doses.

The T-test and the rank sum test were used to test sex-based differences in the main pharmacokinetic parameters in each group. The results showed that the pharmacokinetic parameters of agarotetrol, isoliquiritigenin, piperine, and ferulic acid in rats did not show significant sex-based differences (*p* > 0.05).

After long-term administration, the distribution order of agarotetrol in various tissues of rats was kidney > liver > heart > brain; the distribution order of tissues in the low- and middle-dose groups of isoliquiritigenin was liver > kidney > heart > brain, and that in the high dose group was kidney > liver > heart > brain. The tissue distribution of piperine in each dose group was liver > kidney > heart > brain, and that of ferulic acid in each dose group was kidney > liver > heart > brain. Isoliquiritigenin was not detected in all tissues during the recovery period, and agarotetrol was not detected in the brain, heart, or liver tissues and its concentration in kidney tissues was lower than 2.50 ng ml^−1^. Piperine was not detected in the brain, heart, and kidney, but the concentration in liver tissue was less than 6.95 ng ml^−1^. Ferulic acid was not detected in the brain but was present in the heart, liver, and kidney tissues.

Unlike chemical medicines, TCM often contains many different kinds of medicinal materials, and each medicinal material can also have different ingredients. How to choose ingredients to represent the pharmacokinetics of TCM is a problem faced by the development of TCM. How to guide the clinical usage of TCM is another important problem faced by TCM. According to the current research methods and means, it is of great significance to study the pharmacology and kinetics of TCM, especially of Tibetan medicine. In future research, we should adopt a more scientific and reasonable evaluation method to evaluate the pharmacokinetics of TCM according to its characteristics, to provide a more valuable reference for clinics.

In summary, by adopting and verifying a previously described methodology, the pharmacokinetic characteristics of the classic Tibetan medicine RYZB tablet after single administration and long-term administration were analyzed, which will not only play a guiding role in supporting the clinical application of RYZB, but will also accelerate research into Tibetan medicine theory and its application, thus, providing a solid foundation for the acceptance of Tibetan medicine throughout the world.

## Data Availability

The original contributions presented in the study are included in the article/Supplementary Material; further inquiries can be directed to the corresponding authors.

## Supplementary material

SUPPLEMENTARY FIGURE S1 Q1 full-scan mass spectrum of agarotetrol, isoliquiritigenin, piperine, ferulic acid, and internal standard.**(A)** Agarotetrol; **(B)** isoliquiritigenin; **(C)** piperine; **(D)** ferulic acid; **(E)** interior label.

SUPPLEMENTARY FIGURE S2 Ion full scanning mass spectrum of products of agarotetrol, isoliquiritigenin, piperine, ferulic acid, and internal standard.**(A)** Agarotetrol; **(B)** isoliquiritigenin; **(C)** piperine; **(D)** ferulic acid; **(E)** interior label.

SUPPLEMENTARY FIGURE S3 Typical MRM chromatogram of agarotetrol and internal standard in rat plasma by the LC-MS/MS method. I represents the analyte and II represents the internal standard.**(A)** Blank plasma in rats (double blank); **(B)** blank plasma with the internal standard (blank); **(C)** real sample (single administration for 15.0 min); **(D)** quantitative lower limit sample.

SUPPLEMENTARY FIGURE S4 Typical MRM chromatogram of isoliquiritigenin and internal standard in rat plasma by the LC-MS/MS method. I represents the analyte and II represents the internal standard.**(A)** Blank plasma in rats (double blank); **(B)** blank plasma with internal standard (blank); **(C)** real sample (single administration for 15.0 min); **(D)** quantitative lower limit sample.

SUPPLEMENTARY FIGURE S5 Typical MRM chromatogram of piperine and internal standard in rat plasma by LC-MS/MS. I represents the analyte and II represents the internal standard.**(A)** Blank plasma in rats (double blank); **(B)** blank plasma with internal standard (blank); **(C)** real sample (single administration for 15.0 min); **(D)** quantitative lower limit sample.

SUPPLEMENTARY FIGURE S6 Typical MRM chromatogram of ferulic acid and internal standard in rat plasma by LC-MS/MS. I represents the analyte and II represents the internal standard.**(A)** Blank plasma in rats (double blank); **(B)** blank plasma with internal standard (blank); **(C)** real sample (single administration for 15.0 min); **(D)** quantitative lower limit sample.

## Abbreviations

AUC_0∼t_, area under serum drug concentration–time curve (from 0 h to t h); AUC_0-∞_, area under serum drug concentration–time curve (from 0 h to ∞ h); BQL, below the quantization limit; C_max_, maximum serum concentration; ESI, electrospray ionization; LC-HRMS, liquid chromatography high resolution mass spectrometer; LC-MS/MS, liquid chromatography-tandem mass spectrometry; LLOQ, lower limit of quantification; MPA, mobile phase A; MPB, mobile phase B; MRM, multi-reaction monitoring; MRT_0∼t_, mean residence time (from 0 to t h); MRT_0-∞_, mean residence time (from 0 to ∞ h); M–W method, Mann–Whitney U test; QC, quality control; RYZB, Ruyi Zhenbao tablet; t_1/2_, elimination half-life; T_max_, time of maximum concentration; TCM, traditional Chinese medicine.
