# Supplementary figures and images for: Study and exploration of the pharmacokinetics of traditional Tibetan medicine Ruyi Zhenbao tablets after single and long-term administration
Source: Front Pharmacol. 2022 Sep 29;13:948693. doi: 10.3389/fphar.2022.948693 (PMC9559938; doi:10.3389/fphar.2022.948693)

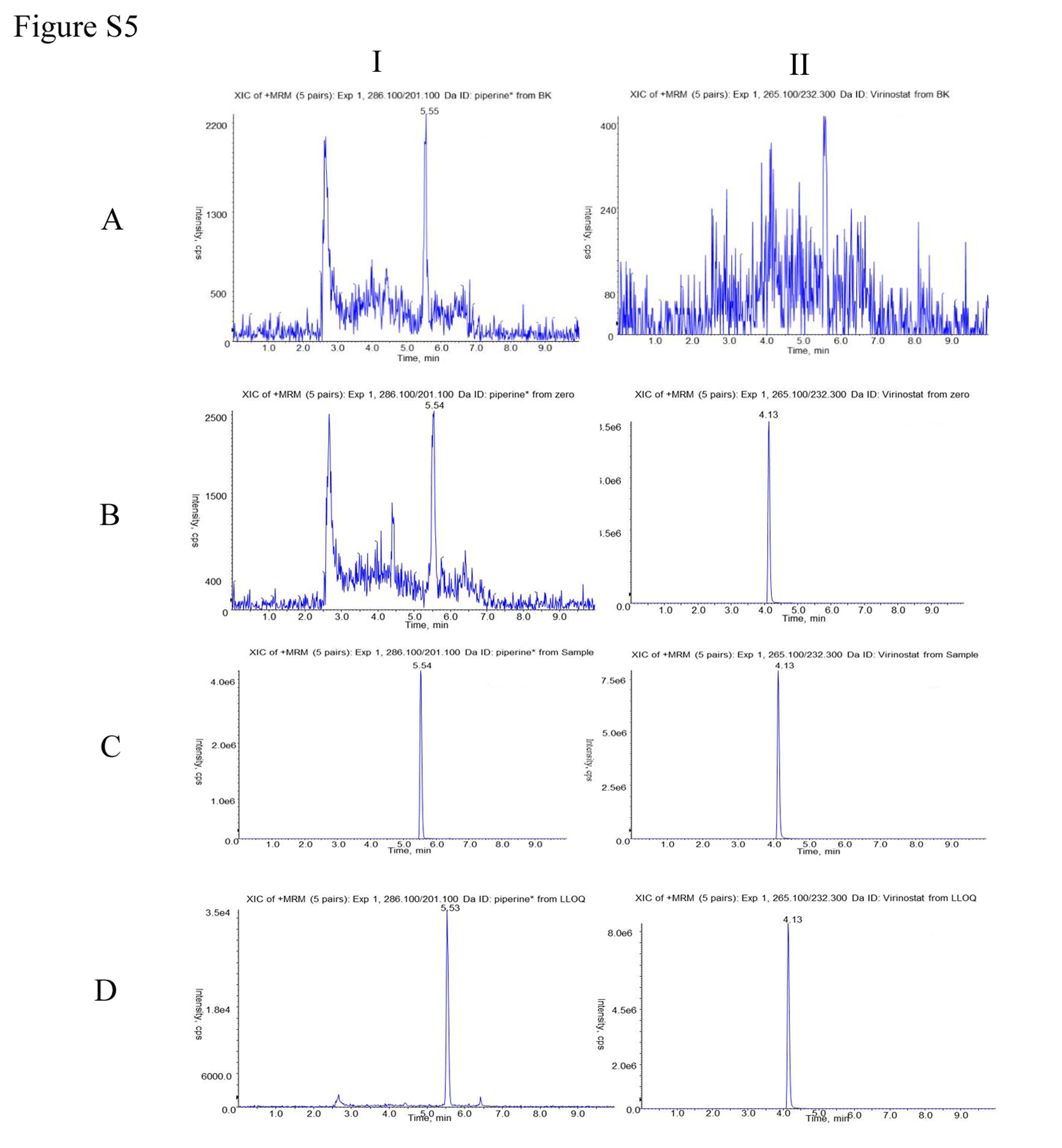

Supplement: Supplementary file 1 [file Image5.jpg]

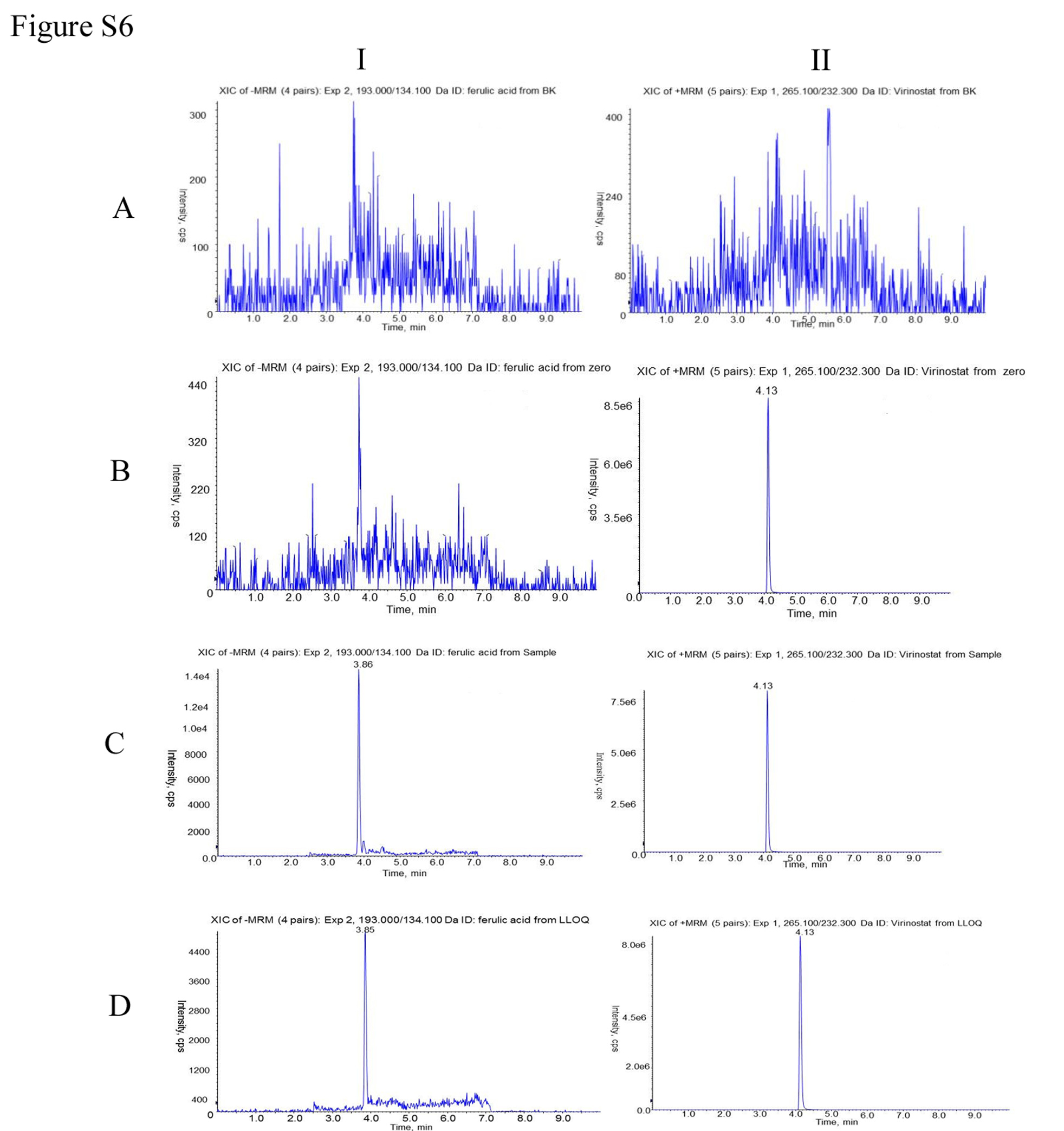

Supplement: Supplementary file 2 [file Image6.jpg]

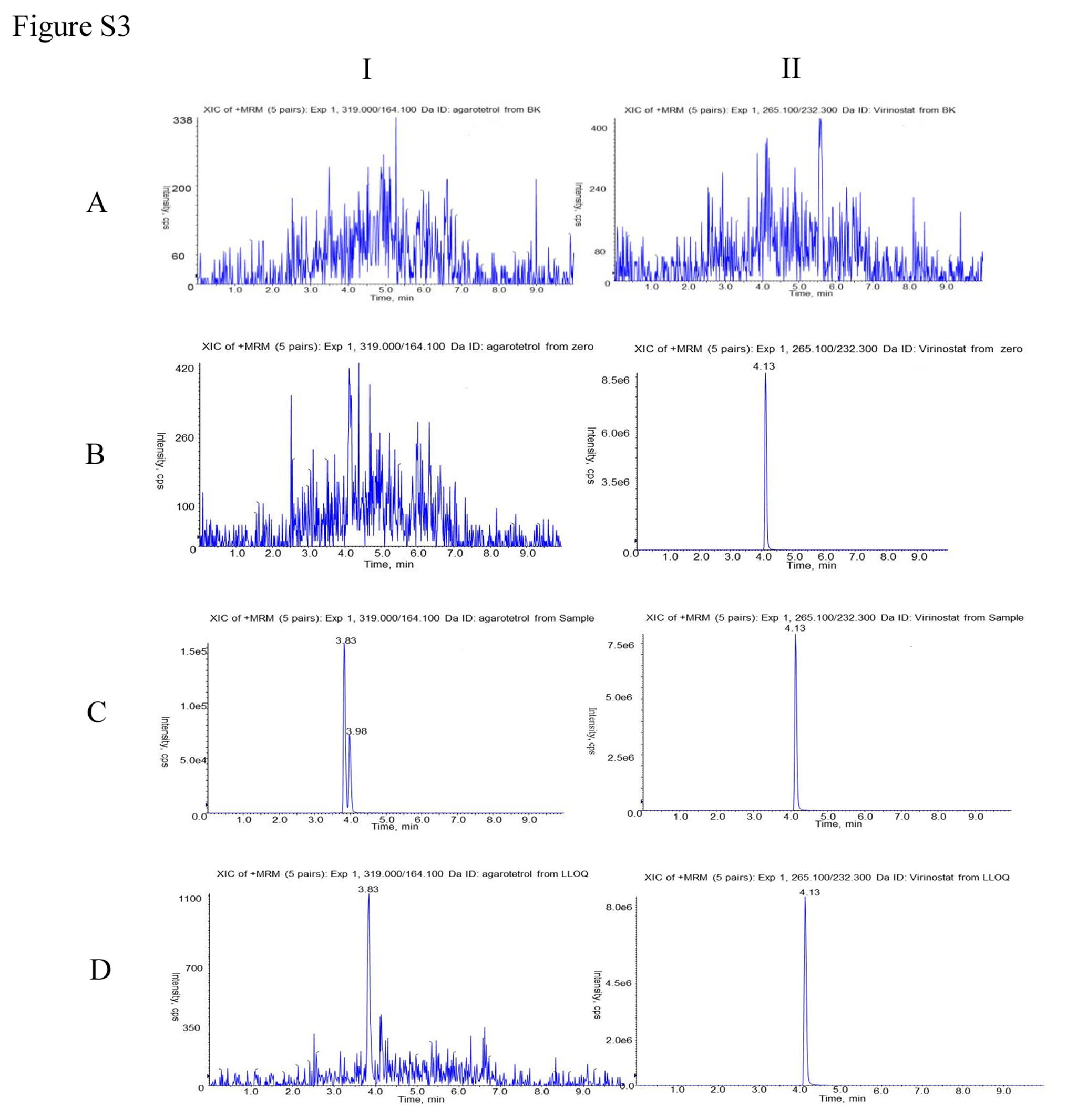

Supplement: Supplementary file 3 [file Image3.jpg]

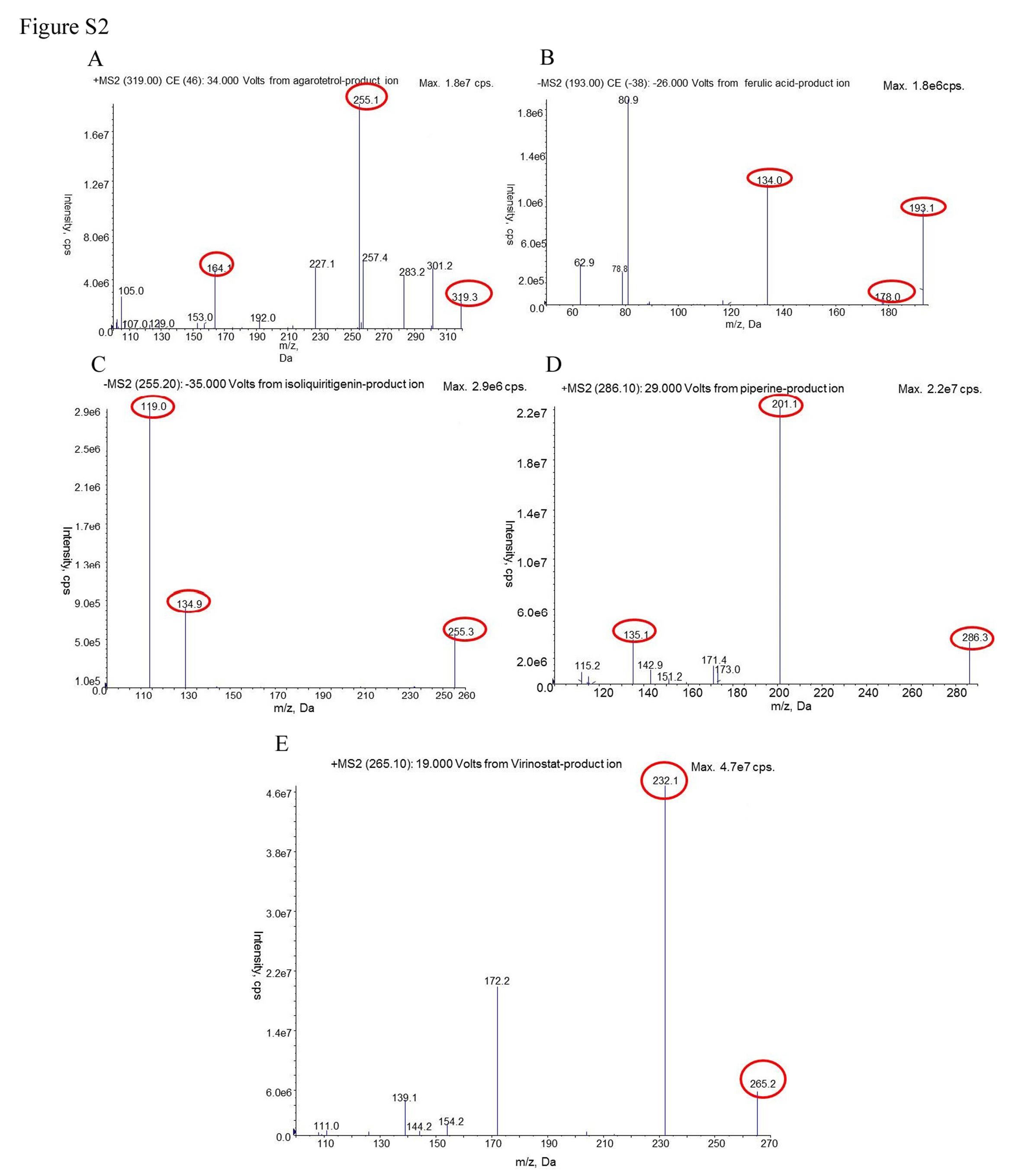

Supplement: Supplementary file 4 [file Image2.jpg]

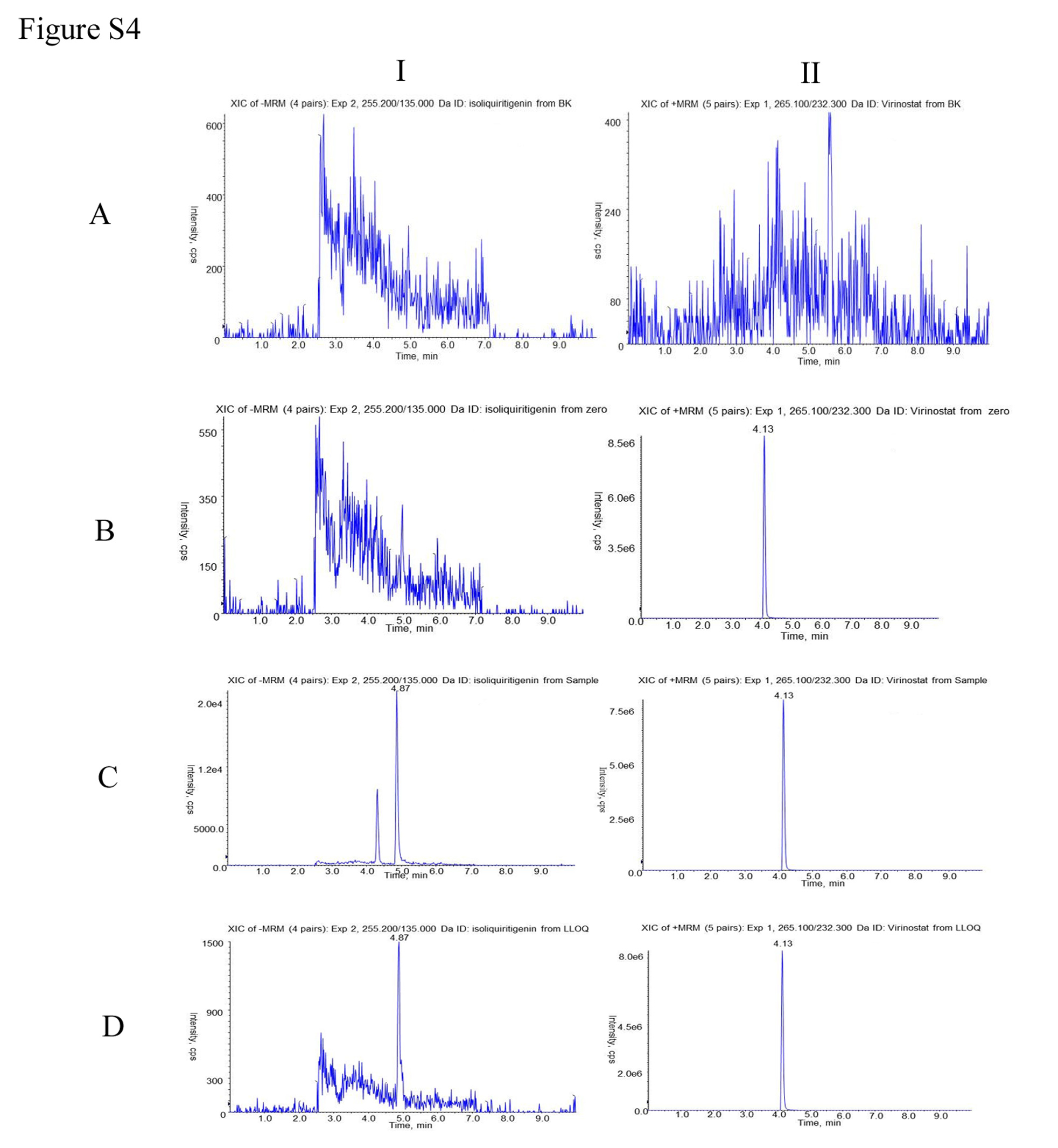

Supplement: Supplementary file 6 [file Image4.jpg]

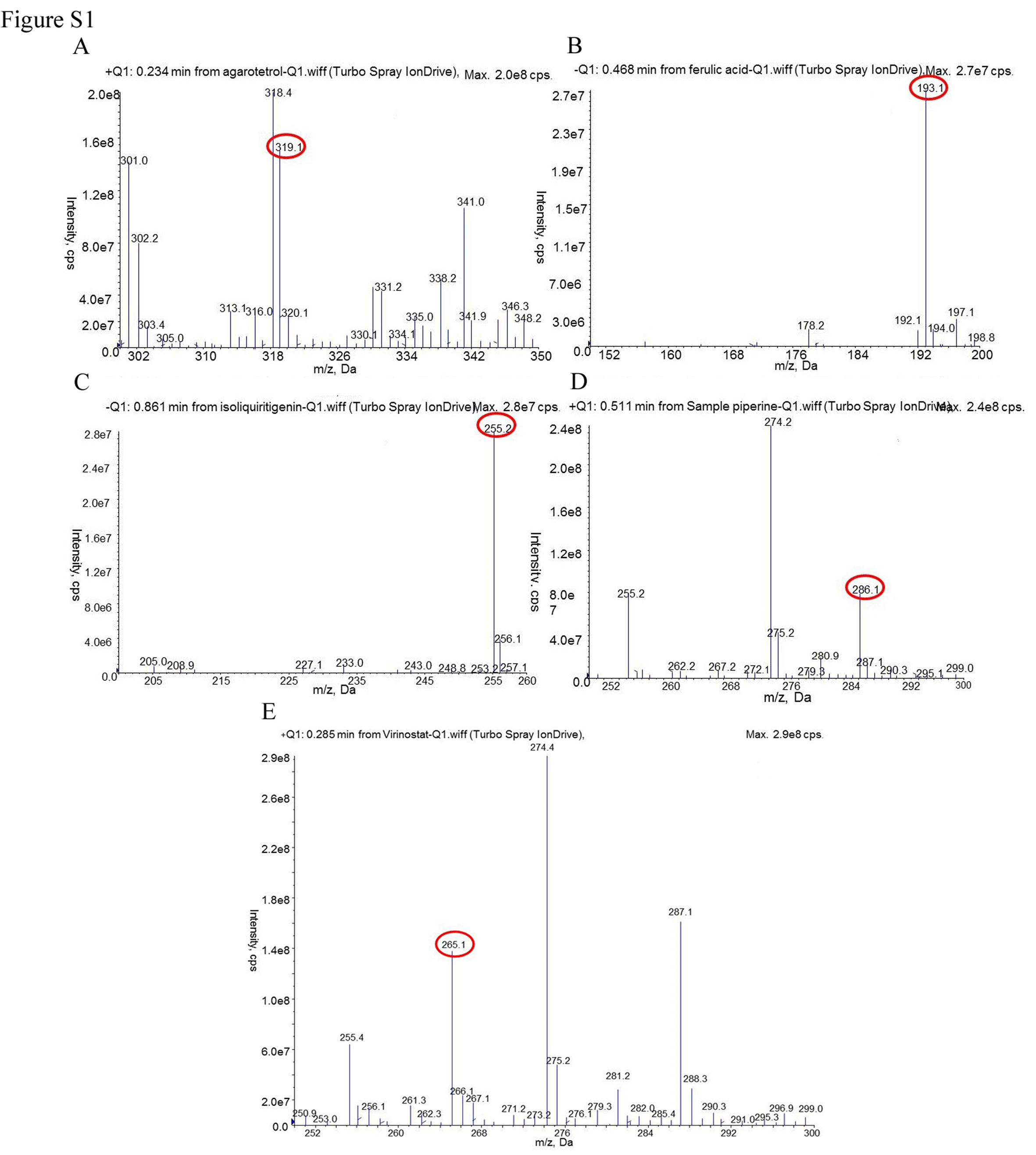

Supplement: Supplementary file 7 [file Image1.jpg]
