# Supplementary material for: Study and exploration of the pharmacokinetics of traditional Tibetan medicine Ruyi Zhenbao tablets after single and long-term administration
Source: Front Pharmacol. 2022 Sep 29;13:948693. doi: 10.3389/fphar.2022.948693 (PMC9559938; doi:10.3389/fphar.2022.948693)
Supplement: Supplementary file 5 [file Table1.doc]

**Table S1 Chromatographic gradient of plasma analysis**

| **Total time (min)** | **Flow rate (µl/min)** | **A%** | **B%** |
| --- | --- | --- | --- |
| 0.0 | 300 | 80.0 | 20.0 |
| 1.00 | 300 | 80.0 | 20.0 |
| 2.00 | 300 | 50.0 | 50.0 |
| 4.50 | 300 | 5.00 | 95.0 |
| 7.00 | 300 | 5.00 | 95.0 |
| 7.10 | 300 | 80.0 | 20.0 |
| 10.0 | 300 | 80.0 | 20.0 |

**Table S2 The mass spectrum conditions of the four components agarotetrol, piperine, isoliquiritigenin and ferulic acid**

| **Compounds** | **Analysis** | **MRM** | **Dwell time^1^ (msec)** | **DP (volts)** | **CE (volts)** |
| --- | --- | --- | --- | --- | --- |
| Agarotetrol | Quantitative | 319.0→164.0 | 80 | 42.0 | 38.1 |
|  | Qualitative | 319.0→255.0 | 80 | 42.0 | 31.7 |
| Piperine | Quantitative | 286.1→201.1 | 80 | 79.0 | 24.0 |
|  | Qualitative | 286.1→135.1 | 80 | 79.0 | 33.0 |
| Isoliquiritigenin | Quantitative | 255.2→135.0 | 80 | -34.0 | -22.0 |
|  | Qualitative | 255.2→118.9 | 80 | -34.0 | -34.0 |
| Ferulic acid | Quantitative | 193.0→134.1 | 80 | -26.0 | -22.0 |
|  | Qualitative | 193.0→178.0 | 80 | -26.0 | -18.0 |
| Internal Standard | - | 265.1→232.3 | 80 | 32.0 | 18.0 |

Note:^1^ Residence time, refers to the time spent each time scanning an ion pair when monitoring the ion pair.

**Table S3 The chromatographic gradients of tissues**

| **Total time (min)** | **Flow rate (µl/min)** | **A%** | **B%** | **Curve** |
| --- | --- | --- | --- | --- |
| 0.00 | 300 | 95 | 5 | 6 |
| 1.00 | 300 | 95 | 5 | 6 |
| 15.0 | 300 | 5 | 95 | 6 |
| 17.0 | 300 | 5 | 95 | 6 |
| 17.1 | 300 | 95 | 5 | 6 |
| 20.0 | 300 | 95 | 5 | 6 |

**Table S4 Method verification results**

| **Validation parameters** | **Results** |
| --- | --- |
| **Selectivity** | The response of interfering components was <20% of the lower limit of the analyte quantification response and < 5% of the internal standard response. |
| **Remained** | The residue in the blank sample should not be >20% of the lower limit of quantification and 5% of the internal standard. |
| **Standard curve and quantitative range** | For 3 analysis batches, at least 75% of the calibration standard samples, the deviation between the calculated concentration and the theoretical concentration of at least 6 calibration samples with effective concentrations was not > 15% (the lower limit of quantification LLOQ should not exceed 20%).The scope as followed: agarotetro, l0.500–1,000 ng/ml; isoliquiritigenin, 0.100–200 ng/ml; piperine, 0.100–200 ng/ml; ferulic acid, 2.50–1,000 ng/ml. |
| **Precision** | Intra-batch:The variation coefficient of the quality control samples should not exceed 15%, and the variation coefficient of LLOQ should not exceed 20%.  Batch: For 3 analysis batches, the coefficient of variation between batches should not exceed 15%, and the coefficient of LLOQ should not exceed 20%. |
| **Accuracy** | Intra-batch:The average accuracy should be within 15% of the marked value of quality control samples, and the LLOQ should be within 20%.  Batch: For 3 analytical batches, the average accuracy should be within 15% of the marked value of quality control samples, and LLOQ should be within 20%. |
| **Dilution reliability** | For high-concentration quality control (QC) after dilution by 5 times, the mean value of the calculated concentration after dilution correction should be within 15% of the marked value, and the precision of the final concentration of all QC samples should not exceed 15%. |
| **Matrix effects** | The coefficient of variation of matrix factors normalized by the internal standard calculated from 6 batches of matrices should not be >15%. |
| **Stability** | The samples were stable in 6 hours at room temperature, within 4 months at -80°C, within 24 hours in automatic sampler after treatment and stable after three freeze-thaw cycles (-80°C, 12 h). The reference stock solution was stable within 2 months at 2–8°C. |

**Table S5 Main pharmacokinetic parameters of agarotetrol after oral administration of RYZB in rats**

| **Dose**  **(g·kg^-1^)** | **Sex** | **t_1/2_ (h)** | **T_max_ (h)** | **C_max_ (ng·mL^-1^)** | **AUC_0-t_ (h·ng·mL^-1^)** | **AUC_0-∞_**  **(h·ng·mL^-1^)** | **MRT_0-t_ (h)** | **MRT_0-∞_ (h)** |
| --- | --- | --- | --- | --- | --- | --- | --- | --- |
| 0.225 | M | 5.23±0.83 | 0.67±0.38 | 32.30±6.86 | 130.52±7.18 | 143.52±17.64 | 4.29±0.90 | 5.73±0.72 |
|  | F | 5.71±3.36 | 1.08±0.80 | 30.70±4.96 | 289.23±209.74 | 302.25±203.16 | 5.50±2.44 | 6.44±2.09 |
|  | Total | 5.47±2.21 | 0.88±0.61 | 31.50±5.43 | 209.21±158.17 | 222.34±155.02 | 4.89±1.77 | 6.09±1.45 |
| 0.450 | M | 5.74±3.04 | 2.00±0.00 | 37.80±9.81 | 274.12±38.18 | 280.12±42.36 | 5.69±0.93 | 6.56±1.78 |
|  | F | 4.59±3.26 | 1.08±0.88 | 46.90±15.30 | 295.02±121.09 | 353.85±120.71 | 4.21±2.38 | 5.34±3.08 |
|  | Total | 5.28±2.77 | 1.54±0.75 | 42.42±12.61 | 284.18±81.36 | 310.36±78.02 | 4.95±1.81 | 6.07±2.10 |
| 0.900 | M | 5.69±3.51 | 2.25±1.64 | 65.51±12.42 | 619.21±187.72 | 702.85±209.25 | 6.35±1.82 | 7.03±3.27 |
|  | F | 7.61±1.78 | 1.58±0.72 | 113.00±41.31 | 918.76±125.48 | 929.08±121.12 | 7.04±2.15 | 7.73±2.61 |
|  | Total | 6.84±2.40 | 1.92±1.19 | 89.50±37.90 | 768.14±217.16 | 838.07±184.09 | 6.69±1.82 | 7.45±2.49 |
| *p* | Dose | 0.491 | 0.150 | 0.000^***^ | 0.000^***^ | - | - | - |
|  | Sex | 0.529 | 0.485 | 0.360 | 0.300 | - | - | - |

Note: t_1/2_: elimination half-life; T_max_: time of maximum concentration; C_max_: maximum serum concentration; AUC_0-t_: area under serum drug concentration–time curve (from 0 h to t h); AUC_0-∞_: area under serum drug concentration–time curve (from 0 h to ∞ h); MRT_0-t_: mean residence time (from 0 h to t h); MRT_0-∞_: mean residence time (from 0 to ∞ h). ^***^*p* < 0.001.

**Table S6 Main pharmacokinetic parameters of isoliquiritigenin after oral administration of RYZB in rats**

| **Dose**  **(g·kg^-1^)** | **Sex** | **t_1/2_ (h)** | **T_max_ (h)** | **C_max_ (ng·mL^-1^)** | **AUC_0-t_ (h·ng·mL^-1^)** | **AUC_0-∞_**  **(h·ng·mL^-1^)** | **MRT_0-t_ (h)** | **MRT_0-∞_ (h)** |
| --- | --- | --- | --- | --- | --- | --- | --- | --- |
| 0.225 | M | 4.36±4.86 | 0.25±0.00 | 0.70±0.03 | 1.32±0.67 | 2.59±2.00 | 2.05±1.22 | 6.21±6.67 |
|  | F | 12.42±7.89 | 0.33±0.14 | 0.48±0.18 | 2.47±2.44 | 5.43±3.73 | 4.99±4.01 | 16.73±9.47 |
|  | Total | 8.39±7.09 | 0.29±0.10 | 0.59±0.17 | 1.89±1.72 | 4.01±2.94 | 3.52±3.10 | 11.52±9.03 |
| 0.450 | M | 10.13±7.55 | 0.33±0.14 | 1.19±0.87 | 4.88±6.74 | 6.70±8.02 | 5.98±7.12 | 14.61±11.91 |
|  | F | 6.03±4.81 | 0.25±0.00 | 1.01±0.23 | 2.39±1.44 | 4.32±3.66 | 2.63±1.21 | 8.29±6.24 |
|  | Total | 8.05±6.08 | 0.29±0.10 | 1.10±0.58 | 3.64±4.57 | 5.51±5.73 | 4.31±4.92 | 11.53±9.16 |
| 0.900 | M | 10.27±6.95 | 0.25±0.00 | 5.63±1.38 | 23.33±8.74 | 24.94±8.93 | 6.90±4.64 | 13.02±8.03 |
|  | F | 7.07±4.19 | 0.50±0.43 | 7.21±2.53 | 22.24±4.18 | 39.82±3.07 | 4.35±2.15 | 8.91±4.74 |
|  | Total | 8.65±5.42 | 0.38±0.31 | 6.42±2.02 | 22.83±6.16 | 29.87±5.97 | 5.63±3.52 | 11.01±6.32 |
| *p* | Dose | 0.971 | 0.990 | 0.000^***^ | 0.000^***^ | - | - | - |
|  | Sex | 0.969 | 0.666 | 0.900 | 0.870 | - | - | - |

Note: t_1/2_: elimination half-life; T_max_: time of maximum concentration; C_max_: maximum serum concentration; AUC_0-t_: area under serum drug concentration–time curve (from 0 h to t h); AUC_0-∞_: area under serum drug concentration–time curve (from 0 h to ∞ h); MRT_0-t_: mean residence time (from 0 h to t h); MRT_0-∞_: mean residence time (from 0 to ∞ h). ^***^*p* < 0.001.

**Table S7 Main pharmacokinetic parameters of piperine after oral administration of RYZB in rats**

| **Dose**  **(g·kg^-1^)** | **Sex** | **t_1/2_ (h)** | **T_max_ (h)** | **C_max_ (ng·mL^-1^)** | **AUC_0-t_ (h·ng·mL^-1^)** | **AUC_0-∞_**  **(h·ng·mL^-1^)** | **MRT_0-t_ (h)** | **MRT_0-∞_ (h)** |
| --- | --- | --- | --- | --- | --- | --- | --- | --- |
| 0.225 | M | 3.64±1.94 | 0.25±0.00 | 1.31±0.30 | 2.42±1.05 | 4.33±1.15 | 1.82±0.96 | 5.19±2.61 |
|  | F | 14.12±14.20 | 0.33±0.14 | 1.64±1.63 | 2.28±1.57 | 5.76±1.73 | 2.40±0.88 | 19.93±20.42 |
|  | Total | 8.85±10.71 | 0.29±0.10 | 1.47±1.06 | 2.35±1.20 | 5.04±1.53 | 2.11±0.88 | 12.61±15.32 |
| 0.450 | M | 6.04±5.19 | 0.50±0.25 | 3.27±2.67 | 11.91±17.80 | 13.71±19.41 | 3.88±4.59 | 7.42±6.51 |
|  | F | 7.72±3.77 | 0.25±0.00 | 3.82±1.30 | 10.21±7.58 | 26.40±21.21 | 3.29±1.00 | 11.73±7.87 |
|  | Total | 6.88±4.16 | 0.38±0.21 | 3.55±1.90 | 11.01±12.20 | 20.01±19.54 | 3.58±2.99 | 9.58±6.88 |
| 0.900 | M | 6.62±4.65 | 0.33±0.14 | 25.42±6.03 | 98.21±52.32 | 105.01±52.01 | 4.78±2.16 | 6.52±2.74 |
|  | F | 4.06±1.74 | 0.25±0.00 | 48.30±23.00 | 122.32±24.40 | 147.32±40.71 | 3.88±2.08 | 5.71±2.38 |
|  | Total | 5.34±3.44 | 0.29±0.10 | 36.81±19.63 | 110.02±38.81 | 126.31±47.54 | 4.33±1.96 | 6.11±2.34 |
| *p* | Dose | 0.716 | 0.665 | 0.000^***^ | 0.002^**^ | - | - | - |
|  | Sex | 0.340 | 0.436 | 0.720 | 0.810 | - | - | - |

Note: t_1/2_: elimination half-life; T_max_: time of maximum concentration; C_max_: maximum serum concentration; AUC_0-t_:area under serum drug concentration–time curve (from 0 h to t h); AUC_0-∞_: area under serum drug concentration–time curve (from 0 h to ∞ h); MRT_0-t_: mean residence time (from 0 h to t h); MRT_0-∞_: mean residence time (from 0 to ∞ h). ^***^*p* < 0.001; ^**^*p* < 0.01.

**Table S8 Main pharmacokinetic parameters of ferulic acid after oral administration of RYZB in rats**

| **Dose**  **(g·kg^-1^)** | **Sex** | **t_1/2_ (h)** | **T_max_ (h)** | **C_max_ (ng·mL^-1^)** | **AUC_0-t_ (h·ng·mL^-1^)** | **AUC_0-∞_**  **(h·ng·mL^-1^)** | **MRT_0-t_ (h)** | **MRT_0-∞_ (h)** |
| --- | --- | --- | --- | --- | --- | --- | --- | --- |
| 0.225 | M | 54.23±20.14 | 1.50±2.17 | 17.21±4.03 | 429.25±64.14 | 916.47±123.18 | 23.10±7.82 | 71.71±2.36 |
|  | F | 43.70±6.25 | 0.33±0.14 | 25.72±15.01 | 401.69±164.18 | 1250.82±654.41 | 24.20±2.44 | 69.82±1.35 |
|  | Total | 49.01±7.44 | 0.92±1.51 | 21.51±10.90 | 415.10±112.47 | 1080.27±236.99 | 23.61±5.21 | 70.71±1.36 |
| 0.450 | M | 25.32±12.24 | 40.03±13.92 | 15.60±2.15 | 431.37±844.17 | 645.34±105.78 | 31.41±3.19 | 43.22±12.53 |
|  | F | 36.57±13.58 | 32.13±27.62 | 19.11±7.75 | 422.64±384.25 | 704.54±201.72 | 26.12±5.45 | 56.51±21.33 |
|  | Total | 29.62±12.95 | 36.01±20.02 | 17.32±5.44 | 426.17±59.35 | 695.19±126.38 | 28.72±4.92 | 49.81±19.81 |
| 0.900 | M | 113.01±56.34 | 16.31±27.43 | 17.90±2.62 | 370.16±31.64 | 1690.15±43.48 | 28.81±4.27 | 171.00±40.81 |
|  | F | 52.41±25.86 | 0.25±0.00 | 24.31±7.68 | 456.71±91.83 | 806.22±412.07 | 27.23±3.87 | 80.01±35.62 |
|  | Total | 82.67±42.77 | 8.29±19.52 | 21.12±6.22 | 413.82±77.46 | 1250.13±625.15 | 28.04±3.74 | 125.32±64.32 |
| *p* | Dose | 0.300 | 0.039^*^ | 0.600 | 0.910 | - | - | - |
|  | Sex | 0.200 | 0.258 | 0.145 | 0.820 | - | - | - |

Note: t_1/2_: elimination half-life; T_max_: time of maximum concentration; C_max_: maximum serum concentration; AUC_0-t_: area under serum drug concentration–time curve (from 0 h to t h); AUC_0-∞_: area under serum drug concentration–time curve (from 0 h to ∞ h); MRT_0-t_: mean residence time (from 0 h to t h); MRT_0-∞_: mean residence time (from 0 to ∞ h). ^*^*p* < 0.05.
